# A New Model Trypanosomatid, *Novymonas esmeraldas*: Genomic Perception of Its “*Candidatus* Pandoraea novymonadis” Endosymbiont

**DOI:** 10.1128/mBio.01606-21

**Published:** 2021-08-17

**Authors:** Alexandra Zakharova, Andreu Saura, Anzhelika Butenko, Lucie Podešvová, Sandra Warmusová, Alexei Yu. Kostygov, Anna Nenarokova, Julius Lukeš, Fred R. Opperdoes, Vyacheslav Yurchenko

**Affiliations:** a Life Science Research Centre, Faculty of Science, University of Ostrava, Ostrava, Czech Republic; b Institute of Parasitology, Biology Centre, Czech Academy of Sciences, České Budějovice (Budweis), Czech Republic; c Zoological Institute of the Russian Academy of Sciences, St. Petersburg, Russia; d Faculty of Sciences, University of South Bohemia, České Budějovice (Budweis), Czech Republic; e de Duve Institute, Université Catholique de Louvain, Brussels, Belgium; f Martsinovsky Institute of Medical Parasitology, Tropical and Vector Borne Diseases, Sechenov University, Moscow, Russia; Washington University School of Medicine

**Keywords:** Trypanosomatidae, Leishmaniinae, bacterial endosymbiont, genomics, metabolism

## Abstract

The closest relative of human pathogen *Leishmania*, the trypanosomatid *Novymonas esmeraldas*, harbors a bacterial endosymbiont “*Candidatus* Pandoraea novymonadis.” Based on genomic data, we performed a detailed characterization of the metabolic interactions of both partners. While in many respects the metabolism of *N. esmeraldas* resembles that of other Leishmaniinae, the endosymbiont provides the trypanosomatid with heme, essential amino acids, purines, some coenzymes, and vitamins. In return, *N. esmeraldas* shares with the bacterium several nonessential amino acids and phospholipids. Moreover, it complements its carbohydrate metabolism and urea cycle with enzymes missing from the “*Ca.* Pandoraea novymonadis” genome. The removal of the endosymbiont from *N. esmeraldas* results in a significant reduction of the overall translation rate, reduced expression of genes involved in lipid metabolism and mitochondrial respiratory activity, and downregulation of several aminoacyl-tRNA synthetases, enzymes involved in the synthesis of some amino acids, as well as proteins associated with autophagy. At the same time, the genes responsible for protection against reactive oxygen species and DNA repair become significantly upregulated in the aposymbiotic strain of this trypanosomatid. By knocking out a component of its flagellum, we turned *N. esmeraldas* into a new model trypanosomatid that is amenable to genetic manipulation using both conventional and CRISPR-Cas9-mediated approaches.

## INTRODUCTION

Trypanosomatidae is a family of obligate parasitic flagellates that includes the causative agents of some severe human diseases (genera *Leishmania* and *Trypanosoma*), as well as those affecting plants (genus *Phytomonas*) (1, 2). The vast majority of trypanosomatids parasitize insects (3), using them as sole either hosts (monoxenous species) or vectors, transmitting the flagellates to vertebrates or plants (dixenous species) (4). Trypanosomatids and related lineages, represented mostly by free-living forms, constitute the class Kinetoplastea, which, together with marine diplonemids, freshwater euglenids, and anoxic environment-dwelling symbiontids, belong to the phylum Euglenozoa (5, 6). Some members of this extremely diverse protist group harbor either extracellular or intracellular bacterial symbionts of varied provenience, as they include alphaproteobacteria, betaproteobacteria, gammaproteobacteria, and cyanobacteria, the latter confined to euglenids (6). From trypanosomatids, only betaproteobacteria of the order *Burkholderiales* have been described so far; they are products of at least two independent acquisitions. The common ancestor of the cosmopolitan genera *Angomonas*, *Strigomonas*, and *Kentomonas* was invaded by “*Candidatus* Kinetoplastibacterium” of the family *Alcaligenaceae* (7, 8), whereas the monospecific *Novymonas* harbors “*Ca.* Pandoraea novymonadis” of the family *Burkholderiaceae* (9). From several available genomes of “*Ca.* Kinetoplastibacterium,” one can predict that these are well-established symbionts that achieved a high level of mutual adaptation following long-term coevolution. In contrast, the endosymbiotic relationship between *N. esmeraldas* and “*Ca.* Pandoraea novymonadis” appears to be much less stable, as the trypanosomatid host does not strictly control bacterial replication, and therefore, the number of endosymbionts significantly varies from cell to cell (9). Compared to its free-living relatives, the genome of “*Ca.* Pandoraea novymonadis” is substantially reduced in size, exhibiting extensive gene loss, low GC content, and numerous gene rearrangements, revealing that bacteria depend heavily on the host for survival. The recently described unidentified endosymbiont of the plant pathogen Phytomonas borealis also shows signs of an unstable relationship (10).

How do the partners benefit from each other? In the above-mentioned symbiosis between the trypanosomatids of the subfamily Strigomonadinae and “*Ca.* Kinetoplastibacterium,” the long-term coevolution triggered significant changes in morphology, biochemistry, and physiology of both bacteria and their flagellate host. Each protist harbors a single bacterium, which replicates in a synchronous manner with its host, ensuring well-coordinated vertical transmission (11, 12). Compared to other trypanosomatids, Strigomonadinae are endowed with an enlarged mitochondrion, presumably because of their increased energy consumption (8, 13). It was proposed that the lack of cell wall in these bacteria facilitates exchange of metabolites (14), which likely include ATP and phosphatidylcholine from the trypanosomatid side (15), while the bacterium reciprocates by providing purines, polyamines, heme, some vitamins, lipids, and amino acids (16–18). Moreover, the synthesis of certain amino acids requires enzymes of both partners, making their metabolic pathways intricately intertwined (19). In addition to metabolites, some host-encoded proteins are targeted from the host cell cytoplasm into the endosymbiont (20). This is a hallmark of a rather stable and intimate relationship between both partners.

It is now generally accepted that endosymbiotic relationships not only were critical for the emergence of the complexity of eukaryotic cell (21, 22) but also have enormous importance in extant ecosystems (23, 24). Our current understanding of the intricacies of symbiotic relationships between eukaryotes on one side and bacteria or archaea on the other is mostly based on the knowledge derived from a variety of animals and plants (25–27). However, this does not align well with the fact that the diversity of protists that exceeds that of multicellular eukaryotes (28, 29) most likely reflects the diversity of respective endosymbiotic relationships.

One way of changing this situation is to introduce and characterize new protist-endosymbiont systems, which inevitably has to be accompanied by their amenability to reverse genetics. Indeed, there is a scarcity of refined molecular genetic tools that currently significantly limits the dissection of the protist-endosymbiotic relationships. However, once a basic tool kit becomes available for these organisms, they may provide outsized insight into symbiosis, as in the case of the cercozoan Paulinella chromatophora (30, 31). Thanks to the wide range of genetic manipulation methods available for the medically important dixenous genera *Leishmania* and *Trypanosoma*, as well as for some of their monoxenous kin (Leptomonas seymouri [32], Crithidia fasciculata [33], *Phytomonas* sp. [34], Herpetomonas muscarum [35], and Lotmaria passim [36]), we believe that the endosymbiont-bearing trypanosomatids can be turned into model systems. This is already exemplified by one species, Angomonas deanei, which was successfully genetically modified (20).

In this work, we describe the genome and transcriptome analysis of *N. esmeraldas* and characterize the metabolic interactions with its endosymbiont. Moreover, we report the development of a molecular toolbox, both conventional and CRISPR-Cas9 based, which will make it possible to tackle the symbiotic relationships between the flagellate host and its cytoplasmic bacteria.

## RESULTS

### Genome of *N. esmeraldas*.

The *N. esmeraldas* genome assembly contains 1,429 scaffolds with an *N*_50_ of 197,811 nucleotides (nt), a total length of approximately 32 Mbp, and a GC content of 62.7%. The genome annotation yielded 9,299 predicted proteins. These values are comparable to those from other available trypanosomatid genomes. The genome and annotation completeness was assessed by BUSCO, and 73% of the universal eukaryotic genes were identified in the assembly. This score is high compared to those of the reference trypanosomatid genomes, which are between 62.4% in the plant pathogen *Phytomonas* sp. strain Hart1 and 74.9% in the model human pathogen Trypanosoma brucei TREU927.

### Metabolic potential of *N. esmeraldas* and its endosymbiont.

We compared the predicted metabolic capabilities of *N. esmeraldas* with those of the model human pathogen Leishmania major. Both trypanosomatids belong to the subfamily Leishmaniinae (37, 38) and are very similar with respect to 486 sequences of selected core metabolic enzymes (Table S1A). Only a few genes present in L. major, such as those encoding NAD-dependent glutamate dehydrogenase, transketolase, and cytosolic malate dehydrogenase, were not found in *N. esmeraldas*. The absence of the latter two enzymes may be compensated for by their respective orthologues in the endosymbiont. We also noted a large number of genes in the genome of *N. esmeraldas* encoding GP63 proteases and the pteridin/biopterin transporters, which are known virulence factors in *Leishmania* species responsible for human leishmaniases (39, 40).

10.1128/mBio.01606-21.10 TABLE S1 Analyses of *Novymonas esmeral*das. (A) List of 486 metabolic enzymes of L. major and their orthologues in *N. esmeraldas*. (B) *Novymonas esmeraldas* metabolic enzymes not present in L. major. (C) Endosymbiont-specific proteins of “*Ca.* Pandoraea novymonadis.” (D) Proteins of *N. esmeraldas* with a putative glycosomal/peroxisomal targeting signal. (E) List of enzymes of *N. esmeraldas* involved in sugar metabolism. (F) Enzymes of gluconeogenesis. (G) Enzymes of the pyrimidine and purine biosynthesis and purine salvage. (H) Enzymes of the F_o_F_1_-ATP synthase and the mitochondrial electron transport chain and mitochondrial contact site and crista organization system (MICOS). (I) Enzymes of amino acid metabolism. (J) Enzymes of the urea cycle. (K) Enzymes involved in synthesis of vitamins and cofactors. (L) Enzymes involved in lipid metabolism. (M) Enzymes involved in phosphor-lipid metabolism. (N) Genes upregulated in the endosymbiont-free *N. esmeraldas* (ES) compared to the wild-type cells (WT). Criteria: BH-adjusted *P* value of <0.001 and fold change of ≥1.5. (O) Genes downregulated in the endosymbiont-free *N. esmeraldas* (ES) compared to the wild-type cells (WT). Criteria: BH-adjusted *P* value of <0.001 and fold change of ≥1.5. (P) Top 10 GO terms most significantly enriched among genes upregulated in aposymbiotic *N. esmeraldas*. The terms are sorted according to the Fisher's test *P* values; those significantly enriched are in bold (*P*  < 0.01). Only GO terms of the three main categories (biological process, molecular function, and cellular component) are included. Annotated, all genes are annotated with the respective GO term in the whole analyzed genome; Significant, a number of genes in the list of the up-/down-regulated ones are annotated with the respective GO term; Expected, an estimate of the number of genes a node of size “Annotated” would have if the significant genes were to be randomly selected from the gene pool. (R) Top 10 GO terms most significantly enriched among genes downregulated in aposymbiotic *N. esmeraldas*. The terms are sorted according to the Fisher's test *P* values; those significantly enriched are in bold (*P* value < 0.01). Only GO terms of the three main categories (biological process, molecular function, and cellular component) are included. Annotated, a total number of genes annotated with the respective GO term in the whole analyzed genome; Significant, a number of genes in the list of the up-/down-regulated ones are annotated with the respective GO-term; Expected, an estimate of the number of genes a node of size “Annotated” would have if the significant genes were to be randomly selected from the gene pool. (S) Data sets used in phylogenomic analysis. (T) Primers used in this study. Download Table S1, XLSX file, 0.7 MB. Copyright © 2021 Zakharova et al. 2021 Zakharova et al. https://creativecommons.org/licenses/by/4.0/ This content is distributed under the terms of the Creative Commons Attribution 4.0 International license.

Of 9,299 predicted protein-coding genes in the *N. esmeraldas* genome, 871 were not found in L. major. The majority of them are hypothetical, 202 are unique to *N. esmeraldas* (Table S1B), and, curiously, seven are also present in the “*Ca.* Pandoraea novymonadis” genome. Three proteins in the latter category are involved in the synthesis of lysine from diaminopimelate, and one is an enzyme of the urea cycle. Another representative example is a pair of argininosuccinate lyases in both the host and endosymbiont genomes, which despite 50% sequence identity do not have a common origin (data not shown). From the perspective of the “*Ca.* Pandoraea novymonadis” genome, 815 of its protein-coding genes (Table S1C) are endosymbiont specific (not present in the host genome). Below, we discuss several aspects of the *N. esmeraldas* and “*Ca.* Pandoraea novymonadis” metabolisms in detail.

**FIG 1:**
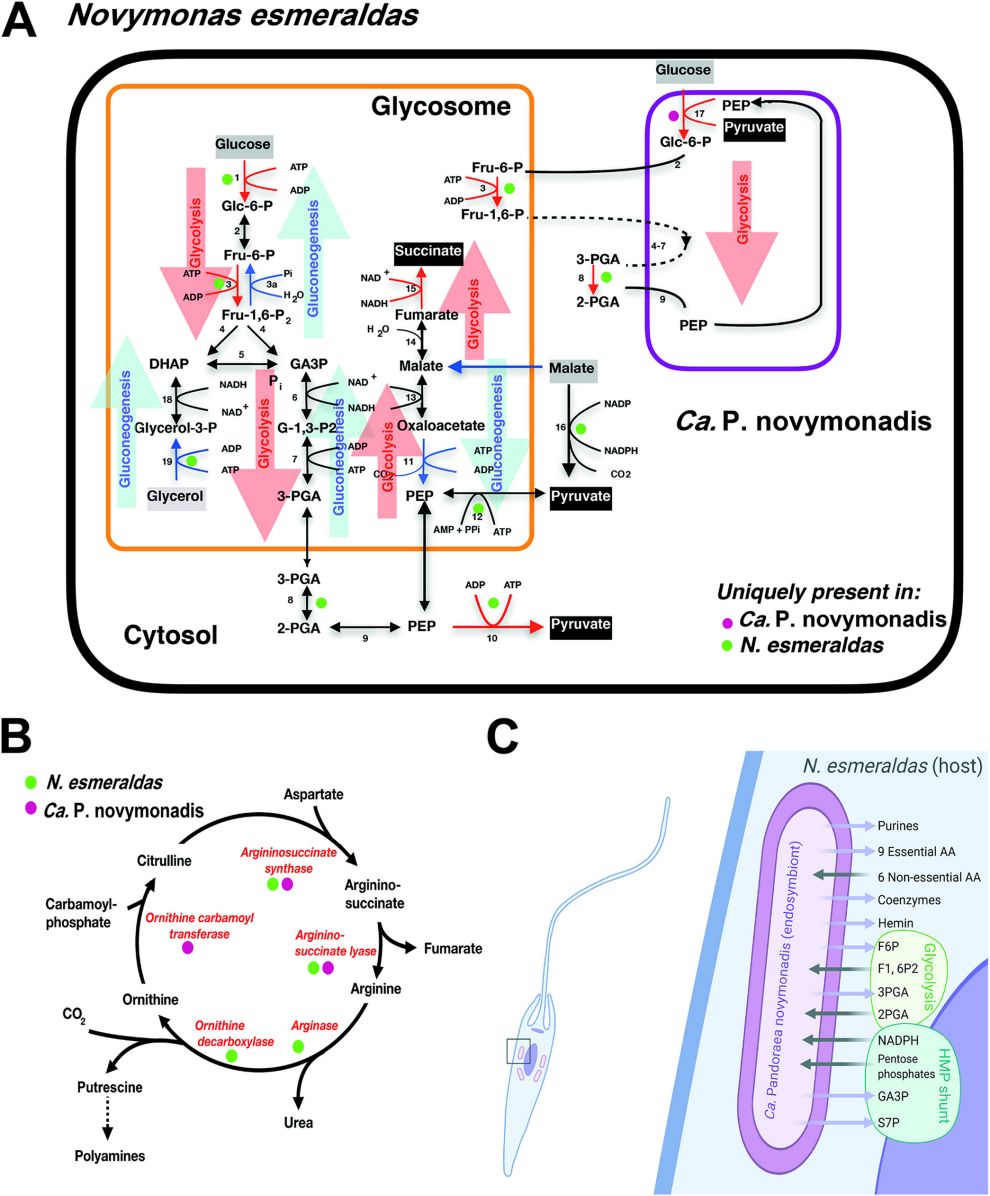
Metabolic exchange between *N. esmeraldas* and its endosymbiont “*Ca.* Pandoraea novymonadis.” (A) Glycolysis and gluconeogenesis. Boxed metabolites are nutrients (in gray) or end products (in black). Glycolysis (red arrows) takes place both in glycosomes and in the endosymbiont. In the latter, exchange of intermediates with the host organism occurs. Gluconeogenesis (blue arrows) takes place exclusively in the host, where malate (resulting from mitochondrial amino acid metabolism) and glycerol (formed from lipid hydrolysis) are converted to glucose 6-phosphate. Enzymes: 1, hexokinase; 2, phosphoglucose isomerase; 3, phosphofructokinase; 3a, fructose-bisphosphatase; 4, fructose-bisphosphate aldolase; 5, triosephosphate isomerase; 6, glyceraldehyde-3-phosphate dehydrogenase; 7, phosphoglycerate kinase; 8, phosphoglycerate mutase; 9, enolase; 10, pyruvate kinase; 11, phosphoenolpyruvate carboxykinase; 12, pyruvate phosphate di-kinase; 13, malate dehydrogenase; 14, fumarate hydratase; 15, NADH-dependent fumarate reductase; 16, malic enzyme; 17, phosphoenolpyruvate-protein phosphotransferase. 18, glycerol-3-phosphate dehydrogenase (NAD); 19, glycerol kinase. Enzyme contributions by host and endosymbiont are indicated by dots of different colors. (B) The urea cycle of *N. esmeraldas*. The urea cycle is divided over host and endosymbiont. Enzyme contributions by host and endosymbiont are indicated by dots of different colors. (C) Summarized scheme of metabolic exchange between *N. esmeraldas* and its endosymbiont “*Ca.* Pandoraea novymonadis.” Abbreviations: F6P, fructose 6-phosphate; F1,6P2, fructose 1,6-bisphosphate; 3PGA, 3-phosphoglycerate; 2PGA, 2-phosphoglycerate; S7P, sedoheptulose 7-phosphate; GA3P, glyceraldehyde 3-phosphate; HMP, hexose-monophosphate shunt, or pentose-phosphate pathway.

**(i) Carbohydrate metabolism.** *Novymonas esmeraldas* has a complete set of the glycolytic pathway enzymes. Several of them carry the peroxisomal/glycosomal targeting signal and, therefore, presumably function in the parasite’s glycosomes (Table S1D). Like most other trypanosomatids, *N. esmeraldas* is capable of using sugars other than glucose, such as galactose, fructose, xylulose, mannose, glucosamine and *N*-acetylglucosamine, as judged by the presence of all enzymes needed for their conversion to fructose-6-phosphate, an intermediate of glycolysis (41) (Table S1E; Fig. 1A). The gluconeogenic pathway, driven by pyruvate-phosphate dikinase, phosphoenolpyruvate carboxykinase, fructose bisphosphatase, and glucose-6-phosphate isomerase, is operational and localized in glycosomes. All genes encoding enzymes of the mitochondrial Krebs cycle were identified, and the classical hexose-monophosphate pathway (HMP), with the exception of transketolase, is present.

The genome of “*Ca.* Pandoraea novymonadis” does not contain hexokinase and pyruvate kinase of the classical glycolytic pathway. Instead, the endosymbiont uses a typical bacterial-type phosphotransferase system (42) that couples the transfer of a high-energy phosphate from phosphoenolpyruvate to the internalization and phosphorylation of hexoses (Fig. 1A). Moreover, since the genes for phosphofructokinase and phosphoglycerate mutase were not found in its genome, “*Ca.* Pandoraea novymonadis” relies on the presence of these three host enzymes to convert hexoses to pyruvate, since the HMP shunt cannot compensate for this gene loss (Fig. 1A). In addition, the endosymbiont’s genome lacks three genes of the first oxidative part of the HMP, namely, glucose-6-phosphate dehydrogenase, 6-phospho-gluconolactonase, and 6-phospho-gluconate dehydrogenase. Interestingly, the transketolase gene is missing from the *N. esmeraldas* genome, making the host and its endosymbiont interdependent in carrying out both the HMP and glycolysis reactions (Table S1F). The presence of genes for fructose-1,6-bisphosphatase in the *N. esmeraldas* and “*Ca.* Pandoraea novymonadis” genomes suggests that gluconeogenesis plays an essential role in both partners, while the bacterium acquires sugar phosphates from the host cell via HMP. Moreover, the trypanosomatid fructose-bisphosphate aldolase and glucose-6-phosphate isomerase allow synthesis of sugars essential for both the bacterium and its eukaryotic host (Fig. 1A).

**(ii) Pyrimidine biosynthesis and purine salvage.** Trypanosomatids (including *N. esmeraldas*) are unable to synthesize their own purines but can salvage them and can synthesize pyrimidines (43, 44) (Table S1G). In contrast, “*Ca.* Pandoraea novymonadis” can synthesize its own pyrimidines and purines, yet it has a limited capacity for the salvage of purine bases and nucleosides. The biochemical pathways for the synthesis of pyrimidines differ in the host and the endosymbiont. While *N. esmeraldas* is endowed with a typical trypanosomatid-like pathway composed of soluble dihydroorotate dehydrogenase and bifunctional orotate phosphoribosyltransferase/orotidine-5-phosphate decarboxylase (45, 46), its endosymbiont harbors a typical bacterial-type pathway (ubiquinone-dependent dihydroorotate dehydrogenase, orotate phosphoribosyltransferase, and orotidine-5-phosphate decarboxylase) (Table S1G).

**(iii) Oxidative phosphorylation and MICOS.** All subunits of F_o_F_1_-ATP synthase and mitochondrial respiratory complexes I through IV were identified in the *N. esmeraldas* genome (Table S1H). Like some other trypanosomatids, *N. esmeraldas* possesses a functional complex I (NADH-ubiquinone oxidoreductase) (47). All subunits of complexes II (succinate dehydrogenase), III (cytochrome *c* reductase), and IV (cytochrome *c* oxidase) are present, although one of the auxiliary subunits of complex II appears to be missing. Its endosymbiont has genes for several subunits of complex I, three catalytic subunits of complex II, and three subunits of complex III (ubiquinol cytochrome *c* reductase, cytochrome *b*, and cytochrome *c*_1_), along with a gene encoding cytochrome *c*. No catalytic subunit and only a single associated subunit of complex IV were identified in “*Ca.* Pandoraea novymonadis.” Nevertheless, the finding of subunits of two ubiquinol oxidase complexes (cytochrome *bo*_3_ and cytochrome *bd*, expressed under high- and low-oxygen conditions, respectively [48]) in its genome implies that at least three of the respiratory complexes contribute to the endosymbiont’s oxidative phosphorylation. A similar situation has been reported for two other symbiont-bearing trypanosomatids, *Angomonas deanei* and Strigomonas culicis (49).

The mitochondrial contact site and crista organization system (MICOS) is a multiprotein complex responsible for crista formation. It is an ancestral eukaryotic protein complex of alphaproteobacterial origin (50). Trypanosomatids are endowed with its divergent form, which was recently carefully characterized in T. brucei (51). All the MICOS proteins were found in the *N. esmeraldas* (but not its bacterial symbiont) genome (Table S1H).

**(iv) Metabolism of amino acids.** *Novymonas esmeraldas* synthesizes nonessential amino acids, along with Thr and Met, and catabolizes amino acids into intermediates of the tricarboxylic acid (TCA) cycle (Table S1I). A few selected examples are described below. Like other Leishmaniinae (but not other trypanosomatids), *N. esmeraldas* synthesizes Arg from citrulline and aspartate, while Gly is split into CO_2_ and formic acid by the mitochondrial glycine-cleavage system, which is lacking in the endosymbiont. The trypanosomatid can salvage Met, as genes encoding all enzymes of the salvage cycle were found in its genome, which, however, does not contain enzymes of the aerobic aromatic amino acid metabolism pathway that convert Phe to fumarate and acetoacetate. Moreover, the flagellate under investigation can neither degrade nor synthesize Lys *de novo*. Among trypanosomatids, only *Leptomonas* and *Crithidia* spp. can convert the bacterial amino acid diaminopimelate to Lys (52). The detection of several diaminopimelate-metabolizing enzymes in both *N. esmeraldas* and its endosymbiont suggests that they have the capacity to perform this biochemical reaction.

**(v) Urea cycle.** Functionality of the urea cycle depends on both symbiotic partners (Fig. 1B). *Novymonas esmeraldas* and its endosymbiont contribute enzymes to the pathway as follows: ornithine carbamoyltransferase is supplied by the endosymbiont, argininosuccinate synthase and argininosuccinate lyase are provided by both host and bacterium, and arginase is supplied exclusively by the host. As a result, the urea cycle metabolites have to shuttle between the host and endosymbiont (Table S1J).

**(vi) Vitamins and cofactors.** Trypanosomatids are obligatory auxotrophs for a number of exogenous cofactors and/or vitamins. They can synthesize neither thiamine (vitamin B_1_) and riboflavin (vitamin B_2_) nor biotin (vitamin B_7_) and cobalamin (vitamin B_12_) (44). However, thanks to the presence of endosymbiont, *N. esmeraldas* can synthesize vitamins B_1_, B_2_, B_6_, and B_7_ but not B_12_ (Table S1K; Fig. 1C). Synthesis of tetrahydrofolic acid (vitamin B_9_) is of special importance for trypanosomatids, since pteridine and folate derivatives are essential cofactors in one-carbon transfer. *Leishmania* is a pteridine auxotroph and has evolved an elaborate and versatile pteridine salvage network capable of accumulating and reducing pteridines (53), which includes biopterin and folate transporters, pteridine reductase, and dihydrofolate reductase-thymidylate synthase (54). *Novymonas esmeraldas* also encodes a battery of enzymes responsible for the uptake of pteridine, its conversion into the dihydrofolic acid, and further utilization in the form of the various folate cofactors.

**(vii) Heme biosynthesis, lipid metabolism, and ROS protection.** Unlike most other trypanosomatids (55), *N. esmeraldas* is able to synthesize its own heme (Table S1K). The components of the heme biosynthetic pathway shared between the host and the endosymbiont are similar to what has been described for two other endosymbiont-harboring trypanosomatids, *A. deanei* and *S. culicis* (49).

Lipid metabolism is virtually the same in *N. esmeraldas* and L. major (Table S1L) (56, 57). The endosymbiont’s capacity for phospholipid metabolism appears to be limited, although, like its host, it can synthesize the ubiquinone required for its respiration (Table S1M).

*Novymonas esmeraldas* encodes catalase, a key component of the reactive oxygen species (ROS) pathway, which is related to its orthologues in other Leishmaniinae (58, 59). No catalase gene was found in the endosymbiont, while both host and endosymbiont have genes for superoxide dismutase, the presence of which renders “*Ca.* Pandoraea novymonadis” aerobic.

### Gene expression changes in the aposymbiotic *N. esmeraldas*.

**FIG 2:**
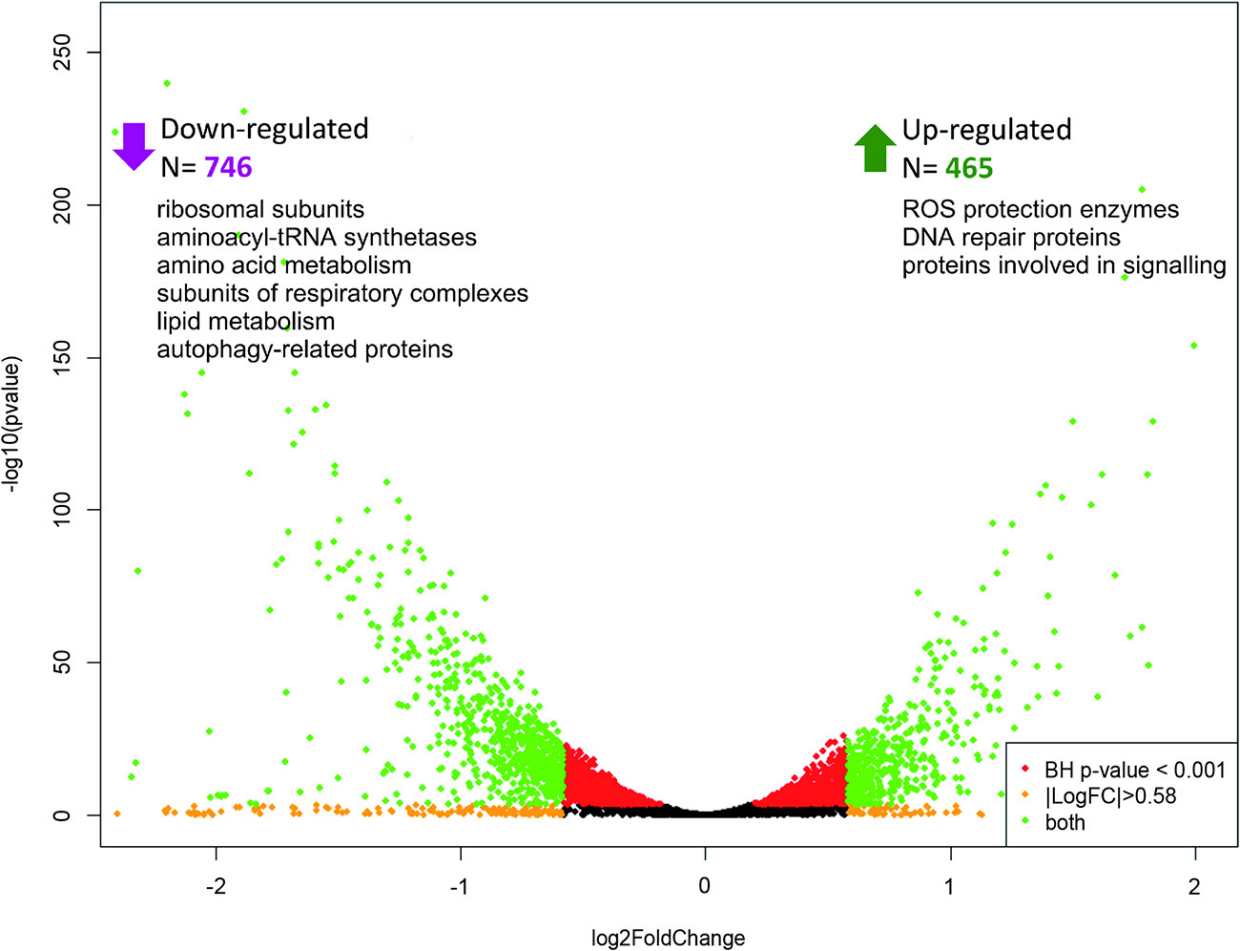
Volcano plot of differentially expressed genes between the E262 and E262-AZI *N. esmeraldas*. The green dots denote significantly differentially expressed genes [BH-adjusted *P* value of <0.001 and fold changes of ≥1.5, or log_2_(fold change) of ≥0.58].

Using the aposymbiotic strain described earlier (42), we identified 1,211 differentially expressed genes (DEGs), of which 465 and 746 were up- and downregulated, respectively, in the aposymbiotic *N. esmeraldas* strain E262-AZI, compared to the wild-type endosymbiont-carrying isolate E262 (1.5-fold change; Benjamini-Hochberg [BH]-adjusted *P* value, <0.001) (Fig. 2; Table S1N and O). The analysis of DEGs was complicated by the lack of functional annotation for ∼42% and ∼34% of up- and downregulated genes, respectively. Nevertheless, as detailed below, the gene ontology (GO) term enrichment analysis sheds light on several aspects of the interactions between *N. esmeraldas* and “*Ca.* Pandoraea novymonadis” (Table S1P and R; Fig. S1 to S7).

10.1128/mBio.01606-21.1 FIG S1 A heat map for 50 genes with the highest variance across samples based on log-transformed expression values. Download FIG S1, PDF file, 0.08 MB. Copyright © 2021 Zakharova et al. 2021 Zakharova et al. https://creativecommons.org/licenses/by/4.0/ This content is distributed under the terms of the Creative Commons Attribution 4.0 International license.

The gene cohort downregulated in the aposymbiotic strain E262-AZI displays a significant enrichment in the GO terms “tRNA aminoacylation for protein translation” and “aminoacyl-tRNA ligase activity,” as well as the presence of a number of ribosomal proteins, multiple aminoacyl-tRNA synthetases, and rRNAs. Thus, the loss of endosymbiont apparently leads to a general decrease of protein biosynthesis in the trypanosomatid host. This may be accompanied by the reduced translation of proteins involved in the interactions at the trypanosomatid-bacterium interface, and/or proteins produced by the host and subsequently imported into the endosymbiont. The aminoacyl-tRNA synthetases downregulated in the endosymbiont-free *N. esmeraldas* include alanyl-, aspartyl-, cysteinyl-, glycyl-, leucyl-, lysyl-, prolyl-, seryl-, and valyl-tRNA charging enzymes. The downregulation of glycyl-, leucyl-, lysyl-, seryl-, and valyl-tRNA may be directly connected to the loss of endosymbionts, which synthetize the corresponding amino acids and provide them to the host (42).

The amino acid metabolism of *N. esmeraldas* also appears to be substantially impacted by the loss of bacteria. This is manifested in a significant decrease in the expression of genes of Gly metabolism (GO term “glycine metabolic process”), including two subunits of the Gly cleavage system (H and P proteins), as well as both cytosolic (NESM_000295500) and mitochondrial (NESM_000597600) hydroxymethyltransferases catalyzing Gly biosynthesis from Ser. The latter observation correlates with the downregulation of the glycyl-tRNA synthetase gene, which facilitates the incorporation of Gly. Moreover, Asp/Asn metabolism is downregulated in the endosymbiont-free *N. esmeraldas*, as reflected by the levels of Asn synthetase, which generates Asn from Asp, and three aspartate aminotransferases, catalyzing the interconversion of Asp and α-ketoglutarate to oxaloacetate and glutamate. In contrast, asparaginase, hydrolyzing Asn to aspartic acid, is upregulated in the endosymbiont-free cells, as is also the case for cysteine desulfurase, which catalyzes Ala biosynthesis from Cys and is involved in Fe-S cluster synthesis. However, other enzymes of the Cys metabolism, such as cystathionine γ-lyase and cystathionine β-lyase, responsible for the breakdown of cystathionine into Cys, α-ketobutyrate, ammonia, and homocysteine and pyruvate, respectively, are downregulated in the aposymbiotic strain. Similarly, the transcription of two genes for branched-chain amino acid aminotransferases, participating in the degradation of Leu, Ile, and Val provided by the endosymbiont (42), and Glu dehydrogenase, an enzyme involved in the metabolism of Glu, is also decreased in these cells. Furthermore, the absence of the endosymbionts causes the downregulation of Phe hydroxylase, catalyzing the conversion of Phe to Tyr, and of S-adenosylmethionine synthetase, responsible for the formation of *S*-adenosylmethionine (AdoMet) from Met and ATP. It appears that the processes of *trans*-methylation and polyamine biosynthesis are affected by the loss of endosymbiont, since AdoMet plays an important role in the respective reactions.

When metabolism is concerned, the loss of endosymbiont leads to a decrease in the mitochondrial respiratory activity. This is reflected by the downregulation of genes falling into the GO categories “ATP synthesis coupled proton transport,” “NAD(P)H dehydrogenase (quinone) activity,” and “mitochondrial inner membrane,” which include multiple subunits of respiratory complexes III, IV, and V (ATP synthase, cytochrome *c* reductase, and cytochrome *c* oxidase). Of note, a decreased expression of several subunits of respiratory complexes was recently reported for aposymbiotic cells of *Strigomonas culicis* (60). However, the situation with glycolysis and gluconeogenesis is less clear. On the one hand, the gene encoding 6-phosphofructo-2-kinase, responsible for the biosynthesis of fructose 2,6-bisphosphate, one of the major regulators of trypanosomatid energy metabolism (61), is significantly upregulated in the aposymbiotic *N. esmeraldas*, similarly to the endosymbiont-free *S. culicis* (60). On the other hand, two other glycolytic enzymes, hexokinase and enolase, along with the gluconeogenic enzyme phosphoenolpyruvate carboxykinase are significantly downregulated.

Lipid metabolism is also repressed in the endosymbiont-free *N. esmeraldas*, as suggested by the downregulation of genes within the GO categories “fatty acid biosynthetic process,” “3-oxo-arachidoyl-CoA synthase activity,” and “very-long-chain 3-ketoacyl-CoA synthase activity,” incorporating multiple fatty acid elongases, malonyl coenzyme A (malonyl-CoA) decarboxylase, β-ketoacyl synthase family protein, delta-5 fatty acid desaturase, 3-ketoacyl-CoA thiolase, isovaleryl-CoA dehydrogenase, 3-ketoacyl-CoA reductase, and enoyl-CoA hydratase.

The loss of the endosymbiont leads to increased ROS production, illustrated by the significant upregulation of genes encoding the enzymes involved in ROS protection, such as trypanothione synthetase, tryparedoxin, tryparedoxin-like proteins, tryparedoxin peroxidase, and glutathione peroxidase (Table S1N). In agreement with this observation, several genes encoding proteins of DNA repair (e.g., mismatch repair proteins MSH3 and MSH6) are also found to be upregulated, probably indicating DNA damage resulting from increased ROS production (Table S1O and R). Similar changes, namely, upregulation of tryparedoxin and genes involved in DNA repair, were reported in *Angomonas deanei* upon removal of the endosymbiont (62).

Sixteen genes encoding proteins associated with autophagy (e.g., autophagy-related protein 8 and ubiquitin-like modifier-activating enzyme ATG7) are significantly downregulated in the aposymbiotic cells. They fall into the “cellular response to nitrogen starvation” and “autophagy” categories and are among genes with the highest variance across samples (Fig. S1). This indicates a reduced requirement for the proteins involved in autophagy, which are likely used for controlling the number of bacteria in the wild-type cells (63). In contrast, genes related to autophagy are upregulated in the aposymbiotic strain of *A. deanei*, compared to the wild type (62).

The GO terms “cAMP biosynthetic process” and “intracellular signal transduction,” assigned to receptor adenylate cyclases, kinases, and a putative methylase, are significantly enriched in the cohort of genes upregulated in the aposymbiotic *N. esmeraldas*. Thus, the loss of the endosymbiont leads to a reduced amino acid, lipid, and carbohydrate metabolism, likely reflecting a significant contribution of “*Ca.* Pandoraea novymonadis” to these processes, as well as reduced needs of the bacterium-deprived trypanosomatid cells (of note, the trend is opposite in *A. deanei*, in which amino acid, lipid, and carbohydrate metabolism are all elevated upon bacterium elimination [62]). On the other hand, upregulation of genes whose products are involved in signaling cascades, ROS protection systems, and DNA repair also accompanies the loss of the bacterial participant of the *Novymonas*-“*Ca*. Pandoraea novymonadis” system.

### *Novymonas esmeraldas* as a new model trypanosomatid.

**FIG 3:**
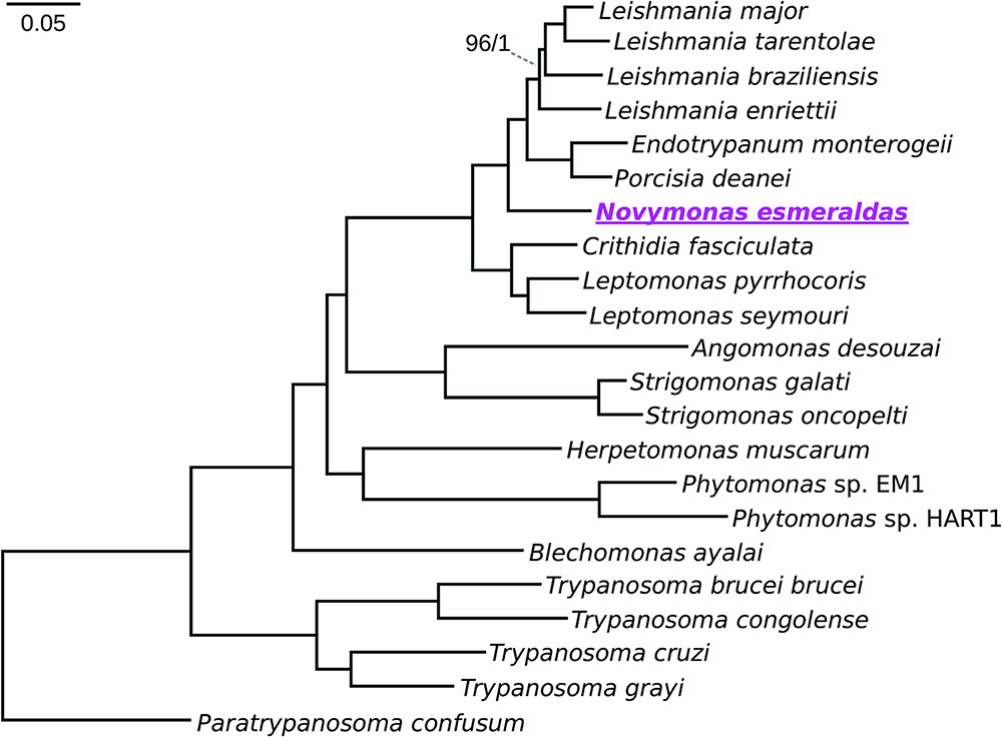
Phylogenetic position of *N. esmeraldas*. Maximum-likelihood phylogenomic tree based on the alignment of 359 proteins encoded by single-copy genes demonstrating the phylogenetic position of *N. esmeraldas* (underlined) as the closest described relative of *Leishmania*. All branches have maximal bootstrap support and posterior probability values (except the branch, where support values are indicated). The bar indicates number of substitutions per site.

We performed a phylogenomic analysis on a set of 640 proteins encoded by single-copy genes in all analyzed kinetoplastid genomes (Table S1S). Both maximum-likelihood and Bayesian trees had the same topology, which is consistent with the results of similar analyses performed earlier (64), and all branches but one displayed maximal statistical support (Fig. 3). *Novymonas esmeraldas* revealed an unambiguous sister relationship with the clade of dixenous Leishmaniinae (*Leishmania*, *Porcisia*, and *Endotrypanum*), confirming the inference based on the sequences of several genes made in the original description of this taxon (9).

**FIG 4:**
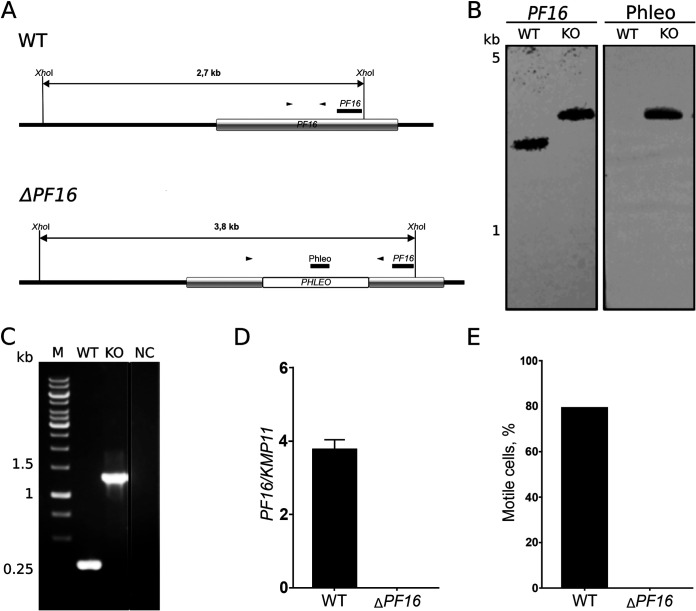
Genetic ablation of *PF16* using CRISPR-Cas9-mediated approach in *N. esmeraldas*. (A) Schematic representation of the wild-type (top) and recombined (bottom) *PF16* alleles of *N. esmeraldas*. Expected sizes of DNA fragments after XhoI digestion and positions of the annealed probes and primers for diagnostic PCR are indicated. (B) Southern blot analysis of the XhoI-digested *N. esmeraldas* genomic DNA of the WT and *PF16*-ablated strains with *PF16* and Phleo probes. (C) Diagnostic PCR for the wild-type and recombined (KO) *PF16* alleles of *N. esmeraldas*. NC, negative control. (D) RT-qPCR control for the wild-type and Δ*PF16* alleles of *N. esmeraldas*. (E) Percent motile cells in the wild type and Δ*PF16 N. esmeraldas*.

The available genomic and transcriptomic data for *N. esmeraldas*, combined with its capacity for genetic modifications, may provide novel insight into the establishment of an endosymbiotic relationship with a bacterium. As a proof of principle that an exogenous DNA can be integrated into the nucleus of *N. esmeraldas*, we first integrated the mCherry-expressing construct into its 18S rRNA locus. The resultant cells exhibited a bright red fluorescence (Fig. S8), indicative of a successful mCherry integration. Next, we set up a CRISPR-Cas9-dependent system for genetic manipulations of this protist. As a template, we used a system previously established in Leishmania mexicana (65), which relies on *FLAG-Cas9* integrated into the same (i.e., the 18S rRNA) locus. Successful integration and expression of *Cas9* were confirmed by PCR (Fig. S9A), reverse transcription-quantitative PCR (RT-qPCR) (Fig. S9B), and Western blotting with anti-FLAG antibodies (Fig. S9C). Of note, the expression of *Cas9* varied significantly between the drug-selected populations, so for downstream experiments, we generated an *N. esmeraldas* population displaying a *Cas9* expression level comparable to that in the L. mexicana transgenic strain (65) (Fig. S9B and C). We noted that *Cas9* expression affects neither cell division (Fig. S9D) nor bacterial load (Fig. S9E). The resultant cells were used to ablate a single-copy gene, *PF16*, encoding a central pair protein of the flagellar axoneme (66). Ablation of this gene in other trypanosomatids makes them invariably immotile (67, 68). The *PF16* homolog of *N. esmeraldas* was disrupted by insertion of the phleomycin resistance gene, and successful integration into both alleles was confirmed by Southern blotting, PCR, and RT-qPCR (Fig. 4A to D). All selected cells were immotile (Fig. 4E), proving that we have established two complementary systems for genetic manipulations in *N. esmeraldas* based on conventional and Cas9-mediated recombination.

10.1128/mBio.01606-21.2 FIG S2 Subgraph representing the 5 most significant GO terms of the category “Molecular Function” (in rectangles) enriched in the group of genes upregulated in aposymbiotic *N. esmeraldas*. Rectangle color represents the relative significance (dark red, most significant). Download FIG S2, PDF file, 0.02 MB. Copyright © 2021 Zakharova et al. 2021 Zakharova et al. https://creativecommons.org/licenses/by/4.0/ This content is distributed under the terms of the Creative Commons Attribution 4.0 International license.

10.1128/mBio.01606-21.3 FIG S3 Subgraph representing the 5 most significant GO terms of the category “Biological Process” (in rectangles) enriched in the group of genes upregulated in aposymbiotic *N. esmeraldas*. Rectangle color represents the relative significance (dark red, most significant). Download FIG S3, PDF file, 0.08 MB. Copyright © 2021 Zakharova et al. 2021 Zakharova et al. https://creativecommons.org/licenses/by/4.0/ This content is distributed under the terms of the Creative Commons Attribution 4.0 International license.

10.1128/mBio.01606-21.4 FIG S4 Subgraph representing the 5 most significant GO terms of the category “Cellular component” (in rectangles) enriched in the group of genes upregulated in aposymbiotic *N. esmeraldas*. Rectangle color represents the relative significance (dark red, most significant). Download FIG S4, PDF file, 0.02 MB. Copyright © 2021 Zakharova et al. 2021 Zakharova et al. https://creativecommons.org/licenses/by/4.0/ This content is distributed under the terms of the Creative Commons Attribution 4.0 International license.

10.1128/mBio.01606-21.5 FIG S5 Subgraph representing the 5 most significant GO terms of the category “Molecular Function” (in rectangles) enriched in the group of genes downregulated in aposymbiotic *N. esmeraldas*. Rectangle color represents the relative significance (dark red, most significant). Download FIG S5, PDF file, 0.03 MB. Copyright © 2021 Zakharova et al. 2021 Zakharova et al. https://creativecommons.org/licenses/by/4.0/ This content is distributed under the terms of the Creative Commons Attribution 4.0 International license.

10.1128/mBio.01606-21.6 FIG S6 Subgraph representing the 5 most significant GO terms of the category “Biological Process” (in rectangles) enriched in the group of genes downregulated in aposymbiotic *N. esmeraldas*. Rectangle color represents the relative significance (dark red, most significant). Download FIG S6, PDF file, 0.08 MB. Copyright © 2021 Zakharova et al. 2021 Zakharova et al. https://creativecommons.org/licenses/by/4.0/ This content is distributed under the terms of the Creative Commons Attribution 4.0 International license.

10.1128/mBio.01606-21.7 FIG S7 Subgraph representing the 5 most significant GO terms of the category “Cellular component” (in rectangles) enriched in the group of genes downregulated in aposymbiotic *N. esmeraldas*. Rectangle color represents the relative significance (dark red, most significant). Download FIG S7, PDF file, 0.04 MB. Copyright © 2021 Zakharova et al. 2021 Zakharova et al. https://creativecommons.org/licenses/by/4.0/ This content is distributed under the terms of the Creative Commons Attribution 4.0 International license.

10.1128/mBio.01606-21.8 FIG S8 Live *Novymonas esmeraldas* cells expressing mCherry (fluorescence microscopy). Bright field, SYTO 24, mCherry and the merged image are presented in the corresponding columns. The mCherry-expressing and wild-type *N. esmeraldas* cells are shown in the upper and lower panels, respectively. Bar, 5 μm. Download FIG S8, PDF file, 0.05 MB. Copyright © 2021 Zakharova et al. 2021 Zakharova et al. https://creativecommons.org/licenses/by/4.0/ This content is distributed under the terms of the Creative Commons Attribution 4.0 International license.

10.1128/mBio.01606-21.9 FIG S9 Establishment of Cas9-expressing *N. esmeraldas.* (A) PCR confirmation of successful integration. Populations 1 to 4 are shown along with the wild type. (B) RT-qPCR of Cas9 expression in populations 1 to 4. Wild-type *N. esmeraldas* and previously established Cas9-expressing L. mexicana were used as negative and positive controls, respectively. (C) Western blotting confirmation of Cas9 expression using anti-FLAG antibodies. Labels are as in panel B. (D) Growth curves of WT and Cas9-expressing *N. esmeraldas*; (E) Bacterial load in WT and Cas9-expressing *N. esmeraldas.* Sizes of DNA (A) and protein (C) fragments are in kilobases and kilodaltons, respectively. Data in panels B and E are summarized from three independent biological replicates. Download FIG S9, PDF file, 0.1 MB. Copyright © 2021 Zakharova et al. 2021 Zakharova et al. https://creativecommons.org/licenses/by/4.0/ This content is distributed under the terms of the Creative Commons Attribution 4.0 International license.

## DISCUSSION

Trypanosomatid flagellates proved instrumental in dissecting numerous features of the eukaryotic cell, such as the replication of mitochondrial DNA, RNA editing, polycistronic transcription, and *trans*-splicing, to name just the best-known examples (1). By turning the easily and affordably cultivable *N. esmeraldas* into a genetically tractable system, we extend its potential to addressing important questions related to endosymbiosis.

There are two endosymbiotic system known in trypanosomatids. The more extensively studied endosymbiosis is the one in the subfamily Strigomonadinae that coevolved for a long time with “*Ca.* Kinetoplastibacterium” This has significantly influenced several morphological features of their hosts, such as the packaging of kinetoplast DNA, the length of the paraflagellar rod, and the extent to which the mitochondrion penetrates the layer of subpellicular microtubules (8, 18, 69). These endosymbionts have a reduced peptidoglycan layer, coordinate their division with that of the host, and are transmitted vertically (70). The presence of these bacteria makes their hosts less fastidious with regard to nutrient intake, relieving the requirement for heme, several amino acids, and some vitamins, which are synthesized by the bacterium (18, 19). The observed close association of endosymbionts with the host’s glycosomes suggests that bacteria may fuel the energy metabolism of the host (71). While the acquisition of bacteria by the ancestor of Strigomonadinae was considered a singular event in the family Trypanosomatidae (72), the *N. esmeraldas*-“*Ca.* Pandoraea novymonadis” system (9) revealed that such a relationship is not exclusive. Less extensive genome reduction and the presence of a complete TCA cycle and amino acid synthesis pathways in the bacterium, combined with a variable number of bacteria per host cell and the use of lysosomes presumably to control their growth, suggested a relatively recent origin of this endosymbiosis. While the genome of “*Ca.* Pandoraea novymonadis” has already passed the period of an intense adaptation to endosymbiosis, the relationships of this bacterium with its host are apparently not as fine-tuned as in the case of “*Ca.* Kinetoplastibacterium” (42, 73).

In this work, we provide a detailed characterization of the metabolic cooperation between *N. esmeraldas* and “*Ca.* Pandoraea novymonadis” (Fig. 1). The bacterium has apparently lost the ability to synthesize polyamines and six amino acids (versus 13 in “*Ca.* Kinetoplastibacterium”), whereas the synthesis of heme, phospholipids, and all vitamins is still preserved (42). In this respect, it is less auxotrophic than the endosymbionts of Strigomonadinae, which lost the ability to synthesize some vitamins and phospholipids (16, 74). In line with this, the aposymbiotic *N. esmeraldas* strain exhibited significant deceleration of growth in the RPMI medium, which was almost fully recovered in the medium supplemented with amino acids, vitamins, and nucleic acid bases (supplemented M199), confirming the nutritional role of the endosymbiont (42). In the Strigomonadinae-“*Ca.* Kinetoplastibacterium” system, the bacteria influence the ability of flagellates to colonize insect hosts (75), regulate the oxygen consumption (76), affect mitochondrial functionality (77), facilitate control of protein synthesis and folding (78), and impact the activity of metalloproteinases (79, 80). Compared to “*Ca.* Kinetoplastibacterium,” “*Ca.* Pandoraea novymonadis” appears to be more self-sufficient for energy generation (42). It also retains more genes involved in cell division, which does not seem to be directly controlled by the host (9).

Previously, we sequenced the genome of “*Ca.* Pandoraea novymonadis” and compared it with those of free-living *Pandoraea* spp. Despite a significant reduction in size and numerous gene losses, it preserves a number of main metabolic pathways, all essential for the trypanosomatid host (42). In contrast to “*Ca.* Pandoraea novymonadis,” the genome of *N. esmeraldas*, analyzed here for the first time, did not experience a substantial reduction and is very similar in size to those of available endosymbiont-lacking trypanosomatids. Indeed, despite the presence of the endosymbiont, almost all genes of the typical trypanosomatid core metabolism have been retained.

The carbohydrate metabolism of *N. esmeraldas* resembles that of L. major and other Leishmaniinae. A complete glycolytic pathway, with part of its enzymes enclosed inside glycosomes (81), is operational. Meanwhile, relying on its own phosphotransferase system to phosphorylate hexose sugars (42), the endosymbiont became dependent on two host enzymes of the pathway, phosphofructokinase and phosphoglycerate mutase, to compensate for the absence of its own corresponding genes (Fig. 1). The same applies to the HMP shunt, interconnecting the host and its endosymbiont. “*Ca.* Pandoraea novymonadis” lacks the first three enzymes (glucose-6-phosphate dehydrogenase, 6-phosphogluconolactonase, and 6-phosphogluconate dehydrogenase) of this pathway, whereas *N. esmeraldas* has lost one of the shunt’s interconverting enzymes, transketolase. Moreover, with the loss of the first part of the HMP shunt, the endosymbiont became dependent on its host for the production of NADPH, required for ROS protection, anabolic reactions, and pentose sugars for the synthesis of nucleotides. *Novymonas esmeraldas* is unable to synthesize its own purines, while its endosymbiont has retained this capacity, with all the genes of the purine *de novo* biosynthetic pathway present in its genome, thus rendering its host completely independent of external purines. Moreover, *N. esmeraldas* can synthetize pyrimidines using trypanosomatid-specific enzymes (46), while its endosymbiont relies on its own set of bacterial-type enzymes to produce these compounds.

The metabolism of amino acids in *N. esmeraldas* is very similar to that described for the other Leishmaniinae (44, 54, 82). Pro, Thr, and Glu serve as important energy substrates which, together with Asp, feed directly into the Krebs cycle, the components of which have been retained in both partner cells. Met is converted via 2-ketobutyrate to succinyl-CoA, and the branched-chain amino acids Ile and Val are oxidized in the mitochondrion to succinyl-CoA and acetyl-CoA. Similarly to the related trypanosomatids, *N. esmeraldas* lacks most of the classical pathway of aromatic amino acid oxidation. Some genes of the anaerobic Phe degradation are present, and aromatic amino acids may be converted to their corresponding aromatic carboxylic acids and alcohols via this pathway. All members of the subfamily Leishmaniinae are auxotrophic for Arg, His, Ile, Leu, Phe, Ser, Trp, Tyr, and Val (44), and the present study confirmed the same feature in *N. esmeraldas*. In order to compensate for this shortcoming, “*Ca.* Pandoraea novymonadis” has retained the ability to synthesize all these amino acids. Conversely, *N. esmeraldas* synthesizes six nonessential amino acids (Ala, Asn, Asp, Cys, Met, and Pro), which its endosymbiont is unable to produce (42).

In order to further dissect the intricately intertwined metabolisms of these endosymbiotic partners and analyze the likely complex targeting of bacterial and host proteins, we have turned *N. esmeraldas* into a novel model trypanosomatid amenable to genetic manipulations by either conventional or CRISPR-Cas9-mediated technology. So far, a similar toolbox developed for the unrelated *Angomonas*-“*Ca*. Kinetoplastibacterium” system was instrumental in tracking the intracellular trafficking of the bacterium-targeted protein (20). Hence, following *P. chromatophora*, *Angomonas deanei* was only the second protist model (and the first one amendable to genetic manipulations) with the capacity to document the emergence of a nonorganellar protein-import machinery.

The important differences documented between the *Angomonas*-“*Ca.* Kinetoplastibacterium” and *Novymonas*-“*Ca.* Pandoraea novymonadis” systems warrant further comparative analyses of their genomes, metabolic pathways, and relationships with bacterial endosymbionts, which will shed light on the evolution of endosymbiosis. The development, refinement, and application of new genetic modification methods will facilitate achieving these goals.

## MATERIALS AND METHODS

### Cultivation, DNA and RNA isolation, and sequencing.

*Novymonas esmeraldas* wild-type strain E262 and its aposymbiotic derivate E262-AZI (42) were cultivated in M199 (Sigma-Aldrich, St. Louis, MO, USA) supplemented with 2 μg/ml hemin (Jena Bioscience, Jena, Germany), 10% heat-inactivated fetal bovine serum (FBS; BioSera Europe, Nuaillé, France), 2 μg/ml biopterin, 100 U/ml of penicillin, and 100 μg/ml of streptomycin (all from Life Technologies/Thermo Fisher Scientific, Carlsbad, CA, USA) at 23°C. Total genomic DNA isolation and sequencing for the E262 cells were reported previously (42). Total RNA was isolated from 5 × 10^7^ cells in three biological replicates using the RNeasy minikit (Qiagen, Hilden, Germany). The cDNA libraries were made and sequenced with 100-nt paired-end reads on the Illumina HiSeq 2000 platform (Macrogen, Seoul, Republic of Korea) as described elsewhere (83).

For genetic manipulations, wild-type cells were cultured at 23°C in brain heart infusion medium (BHI; VWR, Radnor, PA, USA), supplemented with 100 U/ml of penicillin, 100 μg/ml of streptomycin, and 10% heat-inactivated FBS.

### Genome assembly and annotation.

DNA sequencing reads were processed using the BBtools package, v. 36.021 (84). The reads were merged and quality-trimmed using BBmerge with the quality threshold of 20. Nonmerged reads were quality-trimmed using BBduk with the same parameters. The quality of raw and trimmed reads was assessed using FASTQC v.0.11.52 (85). The genome assembly was performed using the SPAdes genome assembler, v. 3.9.0, with default options (86), resulting in 1,429 scaffolds with *N*_50_ of 197,811 nt. The endosymbiont genome (consisting of 6 scaffolds; GenBank accession number MUHY00000000) was extracted from the assembly. The scaffolds shorter than 200 nt and/or with coverage less than 5 were filtered out. The resulting genome assembly contained 1,423 scaffolds with a total length of 32 Mbp.

Prior to assembly, transcriptome sequencing (RNA-seq) reads were subjected to adapter and quality trimming using Trimmomatic v. 0.32 (87) with the following parameters: illuminaclip: TruSeq3-PE-2.fa:2:20:10:8:true; leading: 3; trailing: 3; slidingwindow: 4:15; minlen: 75. All other parameters were left at the default settings. The reads were mapped on the genome using Bowtie 2 (88) with the “–very-sensitive” preset. The transcripts were predicted using StringTie (89) with default parameters. The genome was annotated with Companion (90) using the predicted transcripts, resulting in 8,638 predicted genes and 9,299 predicted proteins. Its completeness was assessed using BUSCO v. 4.1.4 (91).

### Differential gene expression analysis.

The transcriptomes of the endosymbiont-free and wild-type *N. esmeraldas* strains (three independent biological replicates each) were sequenced using the Illumina NovaSeq platform, yielding ∼31 million 150-nt paired-end reads for each of the replicates. The raw reads were adapter and quality trimmed using Trimmomatic v. 0.39 with the following options: TruSeq3-PE-2.fa:2:20: 10; leading: 3; trailing: 3; slidingwindow: 4:15; minlen: 75 (87). The quality control before and after trimming was performed with FastQC v. 0.11.9 (85). Bowtie2 v. 2.4.1 (88) with the “–very-sensitive” option and other parameters left on the default settings was employed for read mapping, with the subsequent read sorting performed using SAMtools v. 1.1 (92). The mapped read counts for each gene were obtained using the featureCounts program with default settings (93). The differential expression analysis was performed using the DESeq2 R package (94). The genes showing over 1.5-fold expression change between E262 and E262-AZI cells with Benjamini-Hochberg-adjusted *P* values below 0.001 were selected for further analyses. Read counts were log-normalized with the DESeq2 rlog function and used for producing a heat map for 50 genes with the highest variance in expression according to the DESeq2 vignette instructions (94).

Gene ontology (GO) terms were assigned to *N. esmeraldas* proteins using PANNZER2 with a PPV threshold of 0.5 (95). GO term enrichment analysis was performed with the topGO R package, v. 2.42.0 (96). Additional functional annotation of differentially expressed genes was performed using BlastKOALA (97). Mitochondrial targeting sequence prediction was carried out with the TargetP-2.0 server (98).

### Phylogenomic and metabolic analyses.

For phylogenomic data set construction, annotated proteins of the kinetoplastid species from Table S1S were clustered using OrthoFinder v. 1.6.12 (99) with default settings. The orthologous groups (OGs) containing proteins encoded by single-copy genes (1,175 OGs in total) were selected for further analysis. The respective protein sequences were aligned using MAFFT v. 7.402 with L-INS-i algorithm (100). At this step, the alignments with <65% average identity were filtered out, resulting in the decrease of the total number of proteins to 359. The remaining alignments were subsequently trimmed using trimAL v. 1.2rev59 (101) with the -strict option and concatenated into a supermatrix of 120,063 amino acid positions used for phylogenomic inferences. A maximum-likelihood phylogenomic tree was inferred with IQ-TREE software v. 1.6.12 (102), automatically selected LG + F + I + G4 model and 1,000 standard bootstrap replicates. A Bayesian tree was constructed using PhyloBayes-MPI v. 1.8 (103) with the LG + CAT model, four discrete Γ rate categories, and removal of invariant sites. Two independent chains ran for 30,000 generations, resulting in good mixing and convergence of all parameters (maxdiff < 0.1 and effective size > 300, estimated after discarding 20% burn-in). The tree topology shown in Fig. 3 became invariant after the first 15 iterations. It was visualized in Mega X v. 10.1.5 (104).

Metabolic pathways were analyzed as in references 44 and 105, using “all against all” BLASTp searches with an E value cutoff 10^−20^. This E value was chosen to discriminate between truly orthologous proteins and more distant homologues, which are not necessarily functional orthologues.

### Genetic manipulations by homologous recombination.

For transfection, E262 cells were grown to a density of 2 × 10^7^ cells/ml. In total, 5 × 10^8^ cells were pelleted at 1,000 × *g* for 15 min and 4°C. Six to eight micrograms of linearized DNA construct was electroporated as described previously with three pulses of 25 μF at 1,500 V (3.75 kV/cm) with a 10-s pause between them (106). Cells were incubated in supplemented BHI without antibiotic for 16 h at 23°C and then under antibiotic selection for 2 to 4 weeks.

To generate a line of *N. esmeraldas* expressing mCherry, this gene was PCR amplified from the p2686 plasmid (107) using the primers mCherry_BglII_F and mCherry_NotI_R (all primers are listed in Table S1T) and cloned into the pF4X1.4neo vector (Jena Bioscience). The resultant plasmid was linearized with SwaI, gel purified, and used for transfection. The transformants were selected in liquid BHI medium supplemented with 200 μg/ml of neomycin (VWR) for 3 weeks.

### Genetic manipulations by CRISPR/Cas9.

The same strategy, previously employed for L. mexicana (65), was adapted for *N. esmeraldas*. The trypanosomatids were transfected with 7 μg of the linearized Cas9/pLEXSYhyg2 plasmid, and the resulting populations were selected in liquid BHI medium supplemented with 200 μg/ml hygromycin. The integration was checked by PCR with the primers SSU_f and Cas9_13_r. Expression of Cas9 was monitored by Western blotting and RT-qPCR with the primers Cas9_10_f and Cas9_5_r. One population with the highest expression of Cas9 was chosen for subsequent experiments.

The U6 promoter, single guide RNA (sgRNA) with trans-activating CRISPR RNA (tracrRNA), and the U6 terminator were amplified using the following primers: PF16KO_Seed_f/Novy_U6_prom_r, Novy _U6_scaffold_f/PF16KO_Seed_r, Novy_U6_term_f/Novy_U6_scaffold_r, respectively. Three fragments were fused using the primers Novy_U6_nested_f and Novy_U6_nested_r and cloned into pLEXSY-neo2.1 (Jena Bioscience). The phleomycin resistance gene was amplified using the primers Spe-Phleo_f and BamHI-Phleo_r and cloned into pLS6-PFR2 (108). The donor DNA sequence was amplified from this plasmid using the primers Donor_PF16KO_f and Donor_PF16KO_r with 30-bp sequences of *PF16* flanking the double-strand DNA break site. The linearized constructs containing the sgRNA under U6 and the PCR product of donor DNA were transfected into *N. esmeraldas*_Cas9 in two transfections, performed one by one. Populations were selected on liquid BHI medium supplemented with an additional 150 μg/ml hygromycin, 200 μg/ml neomycin, and 650 μg/ml phleomycin. Deletion of *PF16* was confirmed by PCR with the primers PF16KO_int_check_f and PF16KO_int_check_r (expected fragment sizes for the wild type [WT] and mutant are 311 and 1,336 bp, respectively), by RT-qPCR with the primers PF16_qPCR_f and PF16_qPCR_r and by Southern blotting with probes for *PF16* and the phleomycin resistance gene. The bacterial load was analyzed by qPCR with the primers GyrA_Pnovy_F and GyrA_Pnovy_R for the bacterial gyrase gene and HKG11_Novy_f and HKG11_Novy_r for a housekeeping gene (*KMP11*) used for normalization.

### Fluorescence microscopy.

Parasites were washed with 1× phosphate-buffered saline (PBS; VWR) and mixed with 40% glycerol solution (vol/vol) in 1× PBS on poly-l-Lys-coated coverslips (Thermo Fisher Scientific). Slides were stained with SYTO 24 green fluorescent nucleic acid stain (Thermo Fisher Scientific), and cells were observed with an Olympus BX-53 microscope (Olympus, Tokyo, Japan) equipped with an Olympus DP73 digital camera. Images were taken with Olympus cellSens v.1.6 software, and merging was done in ImageJ v.1.51n (109).

### Reverse transcription-quantitative PCR.

Cas9 expression was measured by RT-qPCR in the LightCycler480 (Roche Life Science, Penzberg, Germany) as described previously (110, 111) using the following primer pairs: 18S_for/18S_rev, Cas9_10_f/Cas9_5_r, HKG11_Novy_f/HKG11_Novy_r, and PF16_qPCR_f/PF16_qPCR_r. All experiments were performed in biological and technical triplicates, using 18S rRNA (*18S*) and kinetoplast membrane protein-11 (*KMP11*) expression for normalization.

### DNA preparation and Southern blot analysis.

Probes for *PF16* and phleomycin resistance gene were amplified with primers PF16_SB-probe_f/PF16_SB-probe_r and Phleo_SB-probe_f/Phleo_SB-probe_r, respectively, using a PCR digoxigenin (DIG) probe synthesis kit (Roche Life Science). For Southern blotting, 20 μg of DNA was digested with XhoI, separated on a 0.75% agarose gel, and probed as described previously (65, 112).

### Data availability.

The whole-genome shotgun project has been deposited at DDBJ/ENA/GenBank under the accession number JAECZO000000000. The version described in this paper is version JAECZO010000000. The data set used for phylogenomic inferences is available at https://figshare.com/articles/dataset/Novymonas_esmeraldas_phylogenomic_dataset/14618193.
