# Supplementary figures and images for: A New Model Trypanosomatid, Novymonas esmeraldas: Genomic Perception of Its “Candidatus Pandoraea novymonadis” Endosymbiont
Source: mBio. 2021 Aug 17;12(4):e01606-21. doi: 10.1128/mBio.01606-21 (PMC8406214; doi:10.1128/mBio.01606-21)

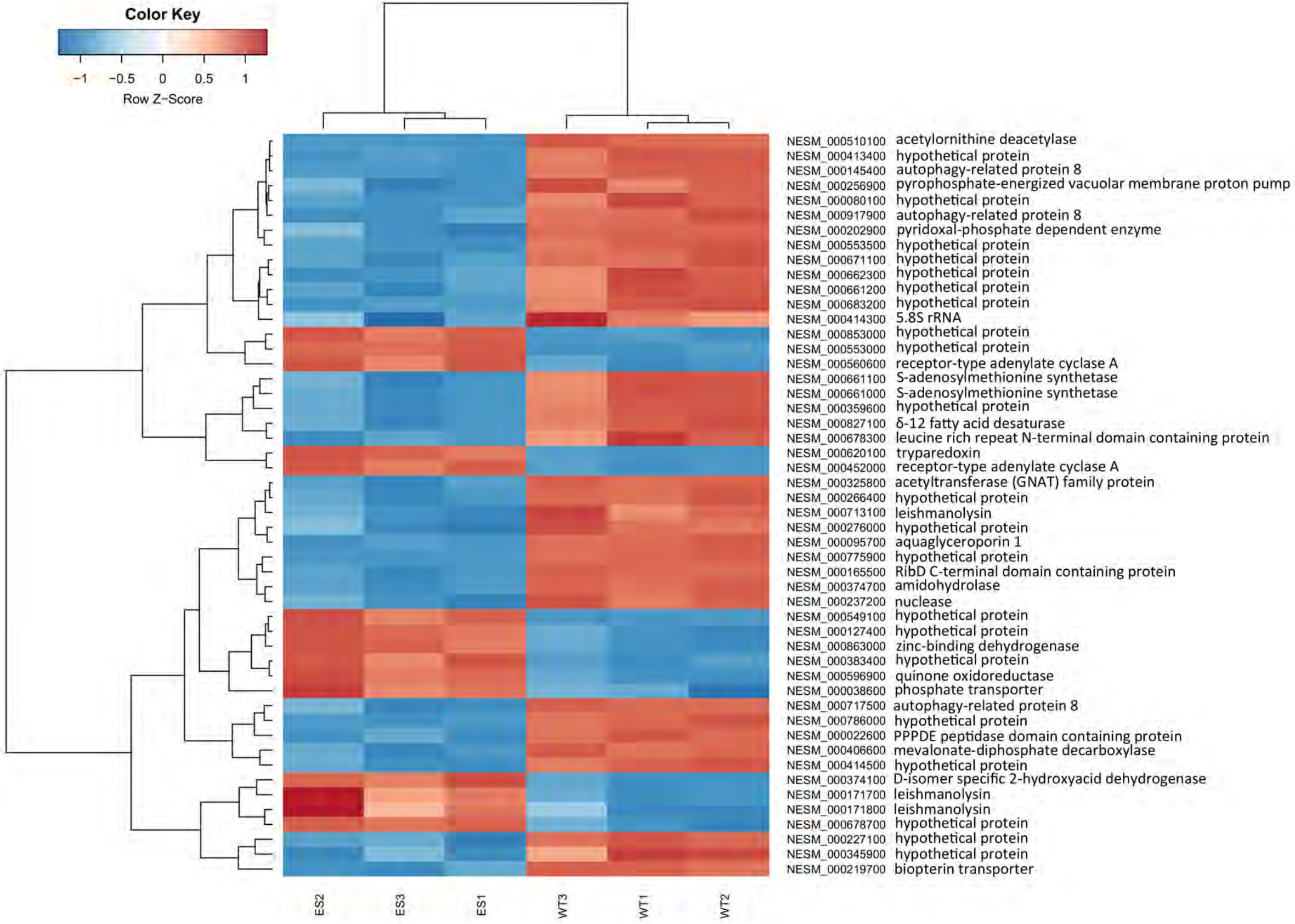

Supplement: FIG S1 [file mbio.01606-21-sf001.pdf]

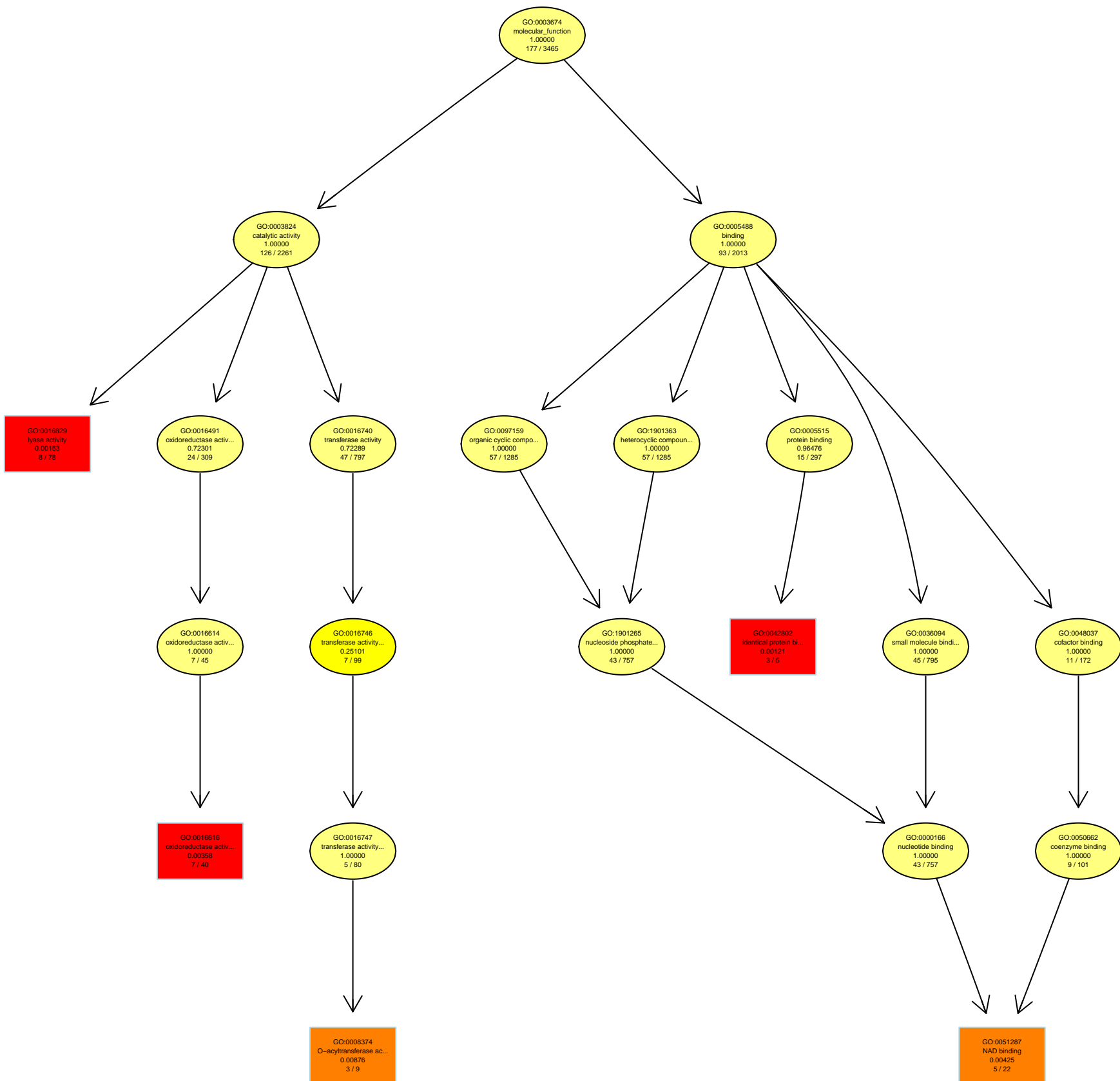

Supplement: FIG S2 [file mbio.01606-21-sf002.pdf]

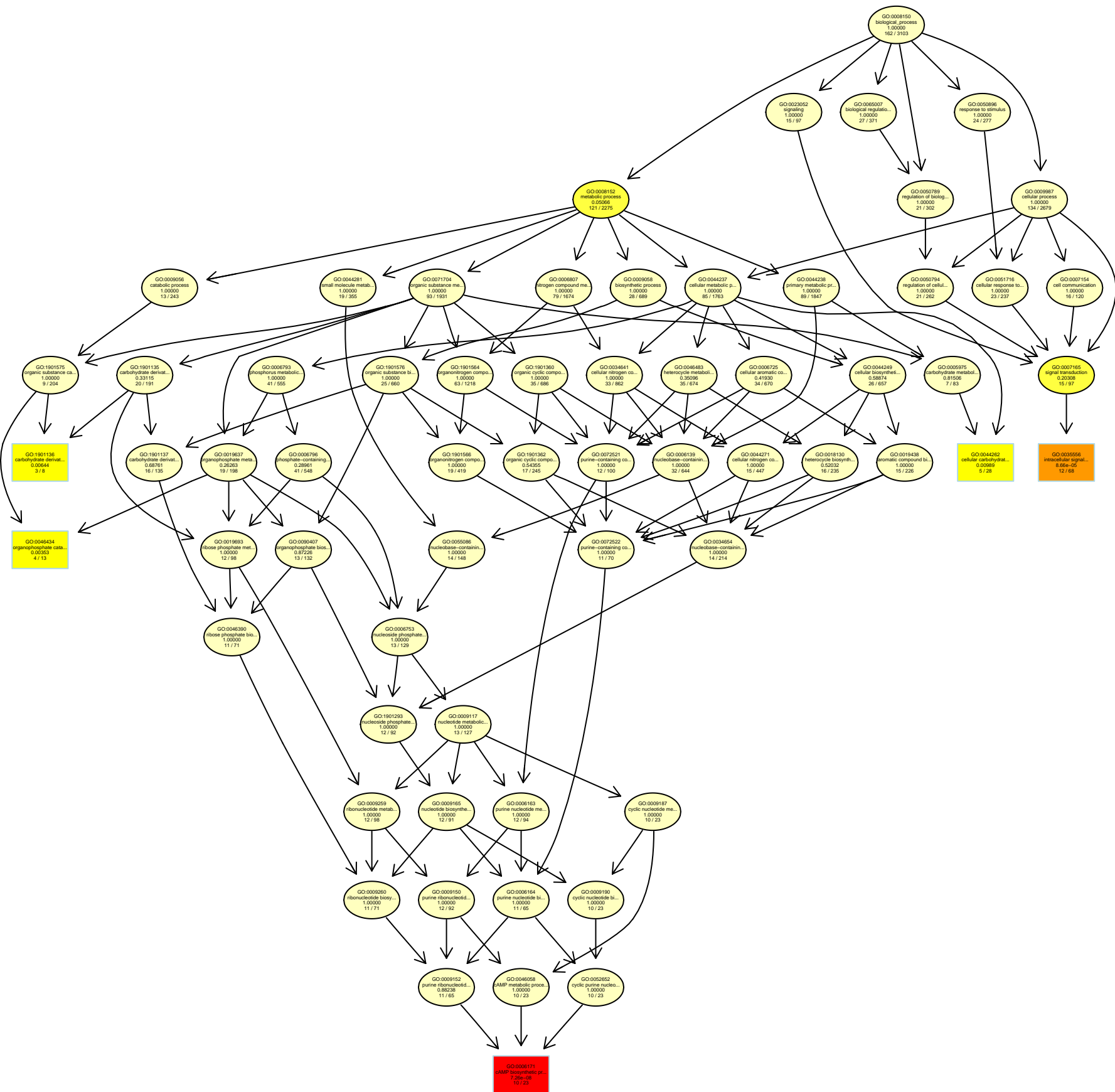

Supplement: FIG S3 [file mbio.01606-21-sf003.pdf]

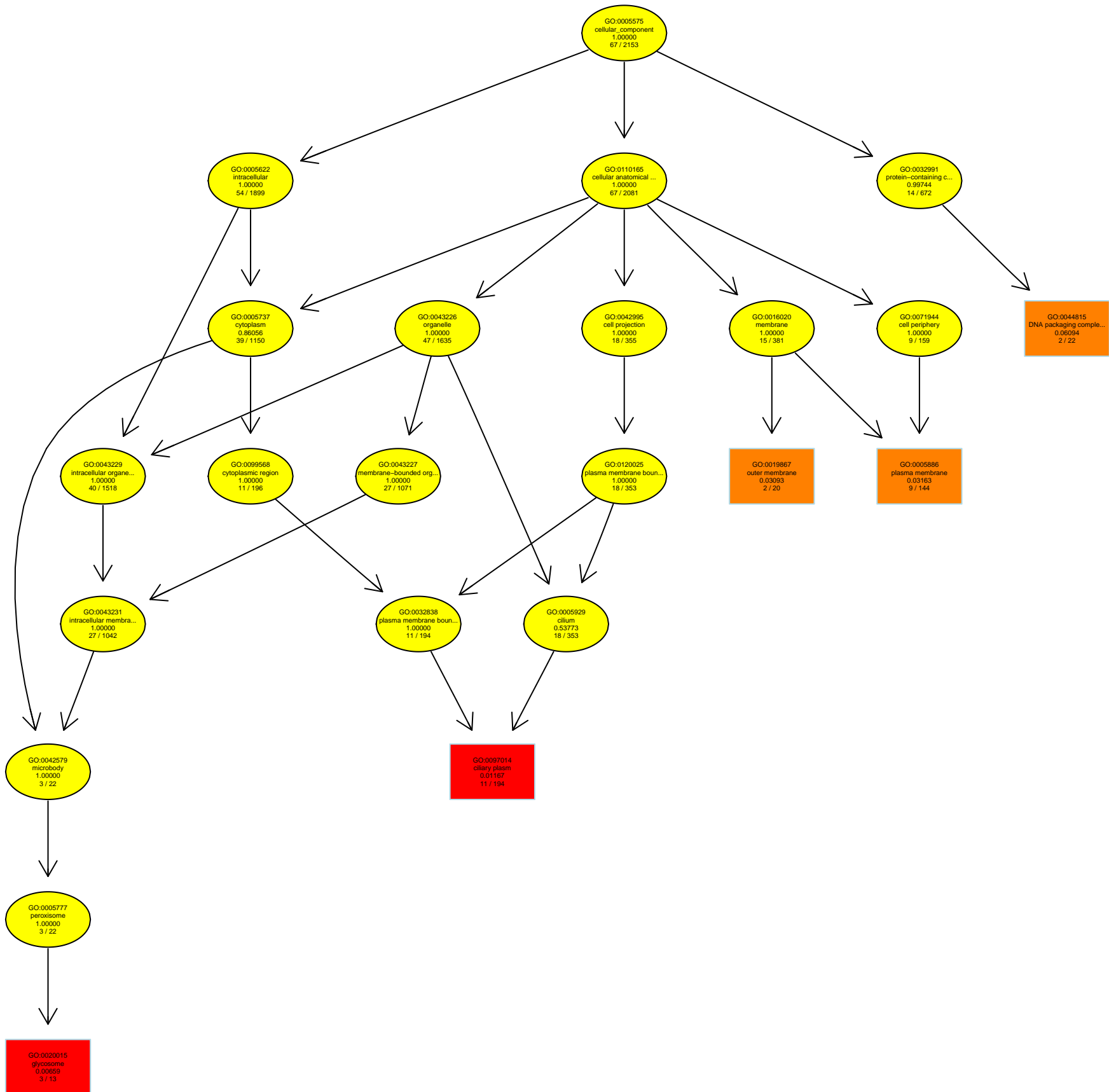

Supplement: FIG S4 [file mbio.01606-21-sf004.pdf]

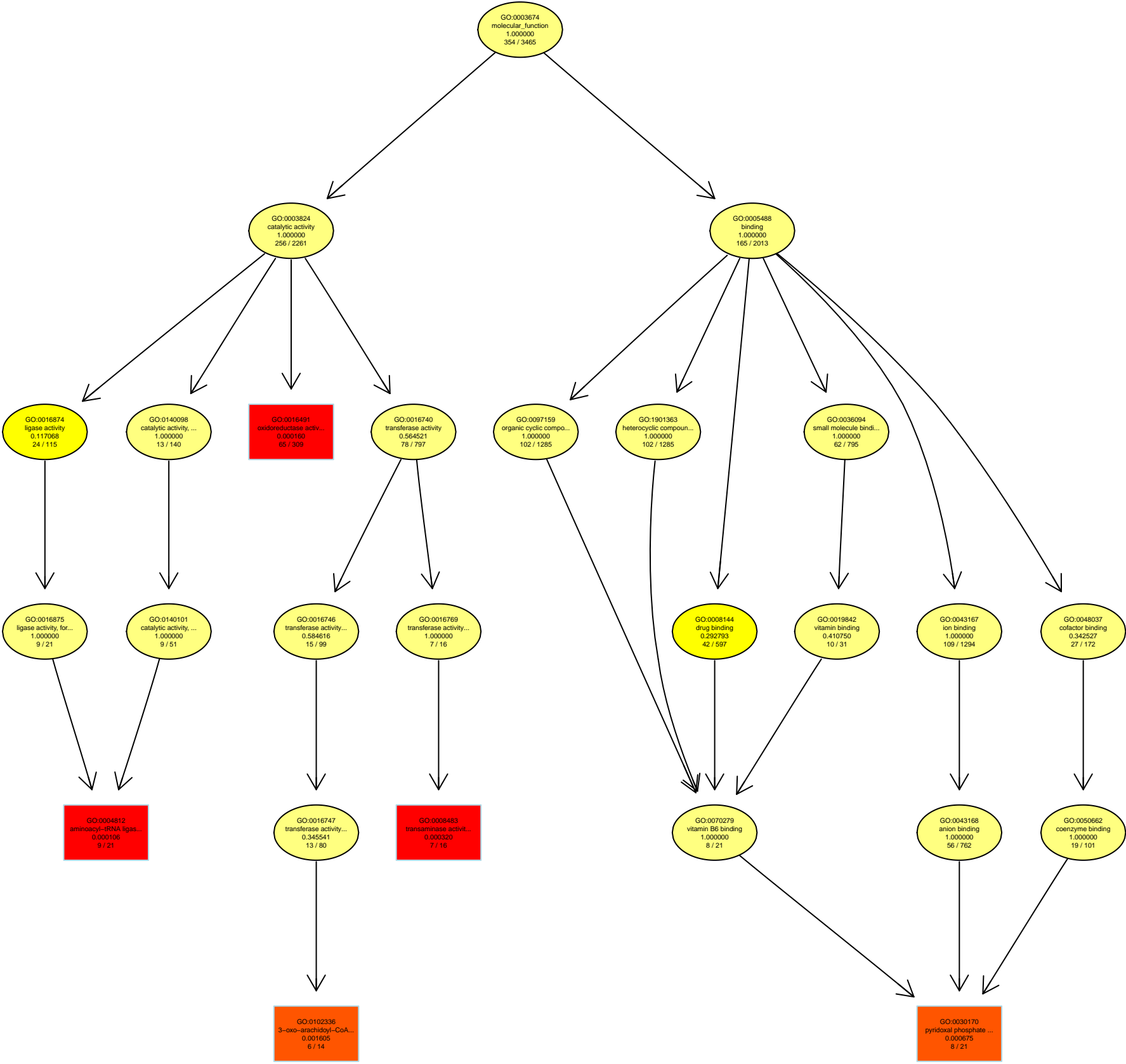

Supplement: FIG S5 [file mbio.01606-21-sf005.pdf]

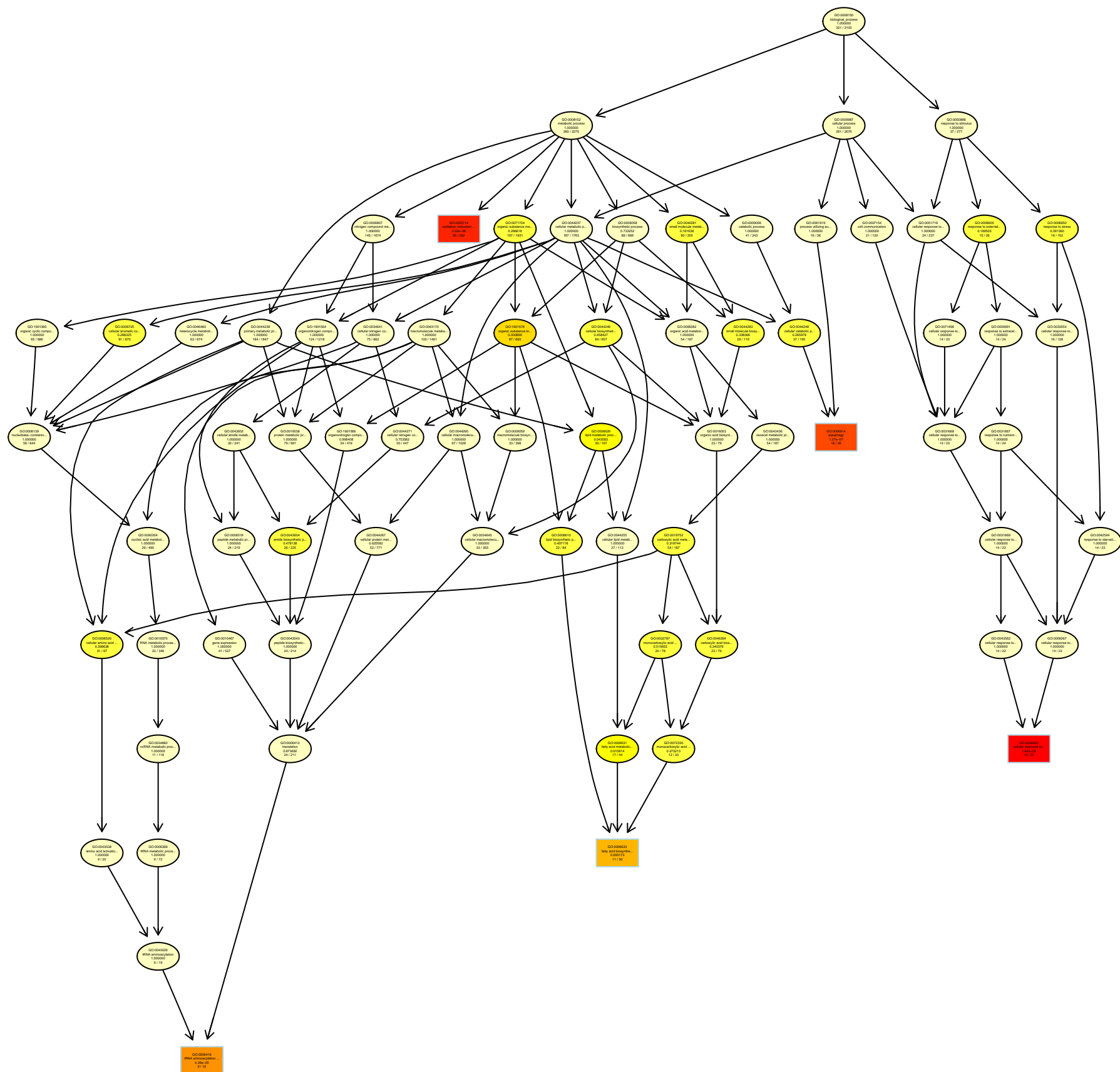

Supplement: FIG S6 [file mbio.01606-21-sf006.pdf]

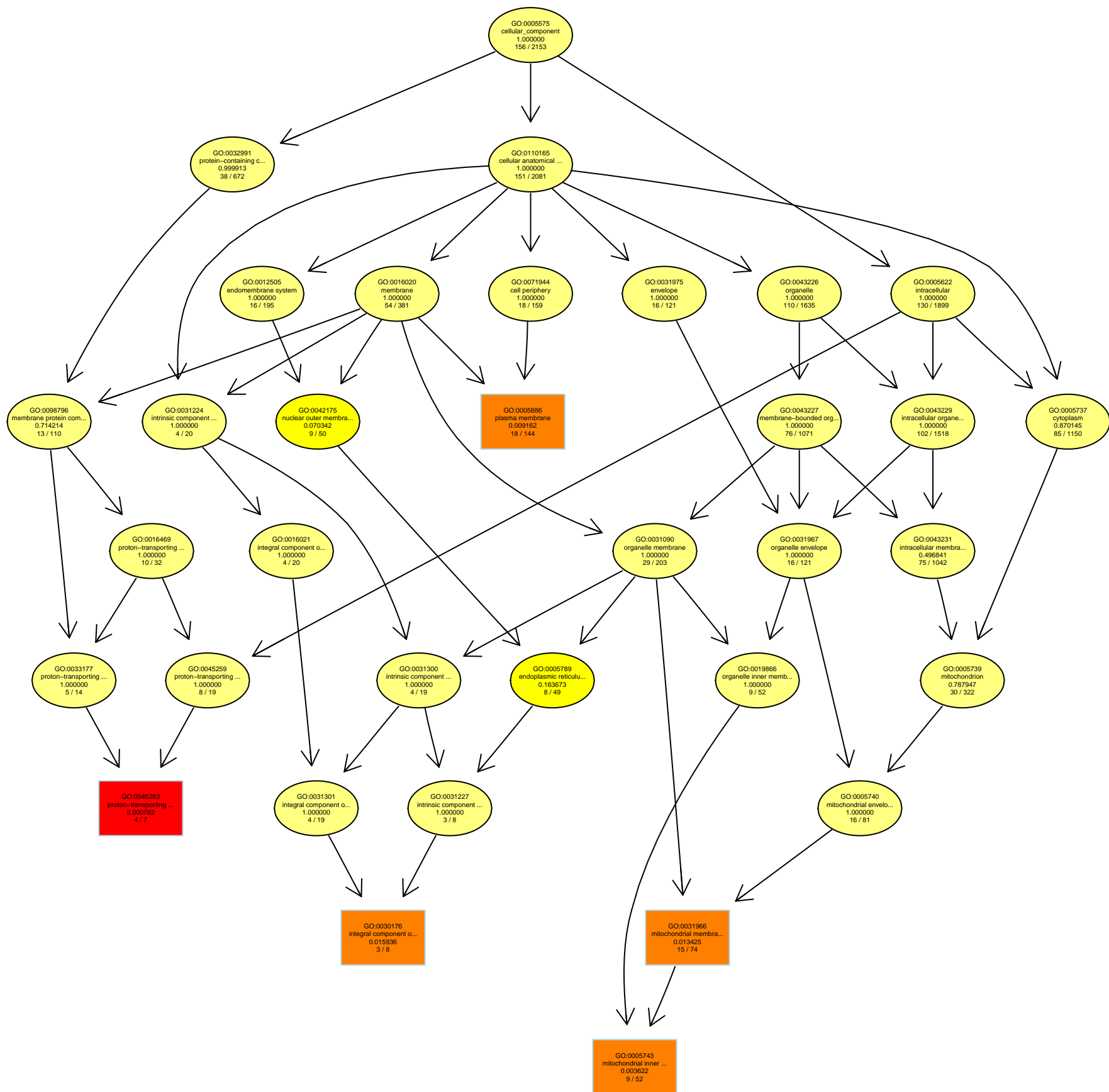

Supplement: FIG S7 [file mbio.01606-21-sf007.pdf]

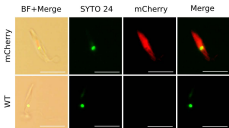

Supplement: FIG S8 [file mbio.01606-21-sf008.pdf]

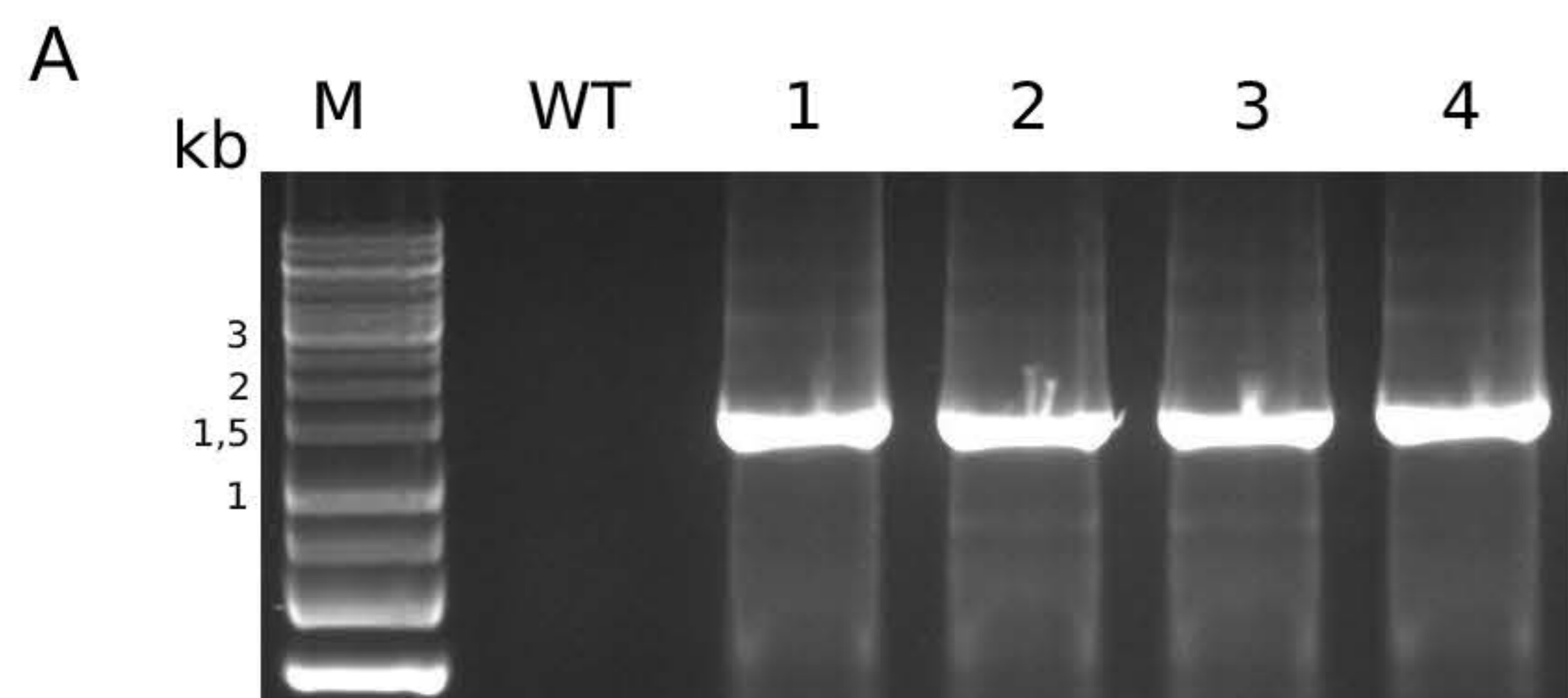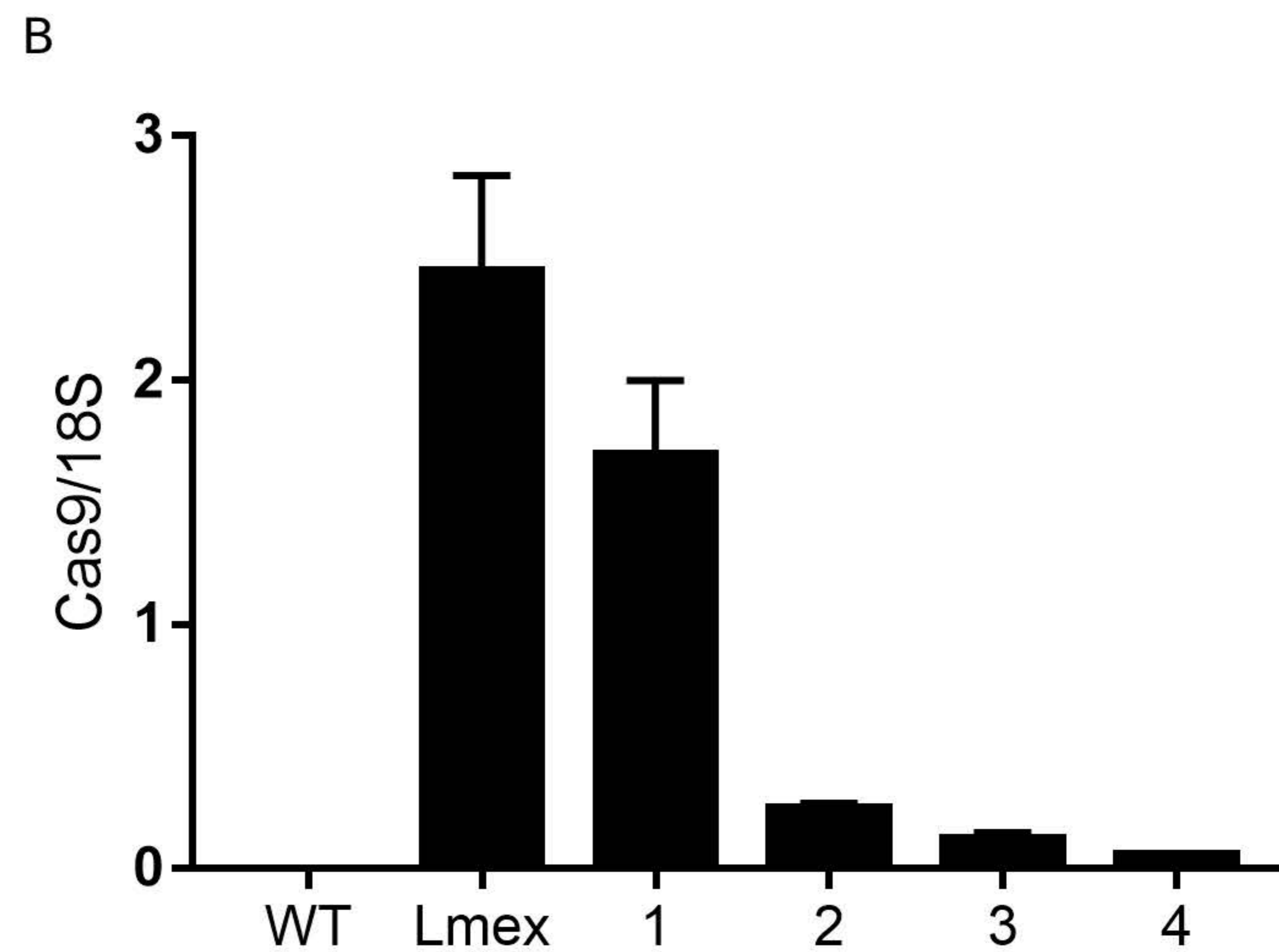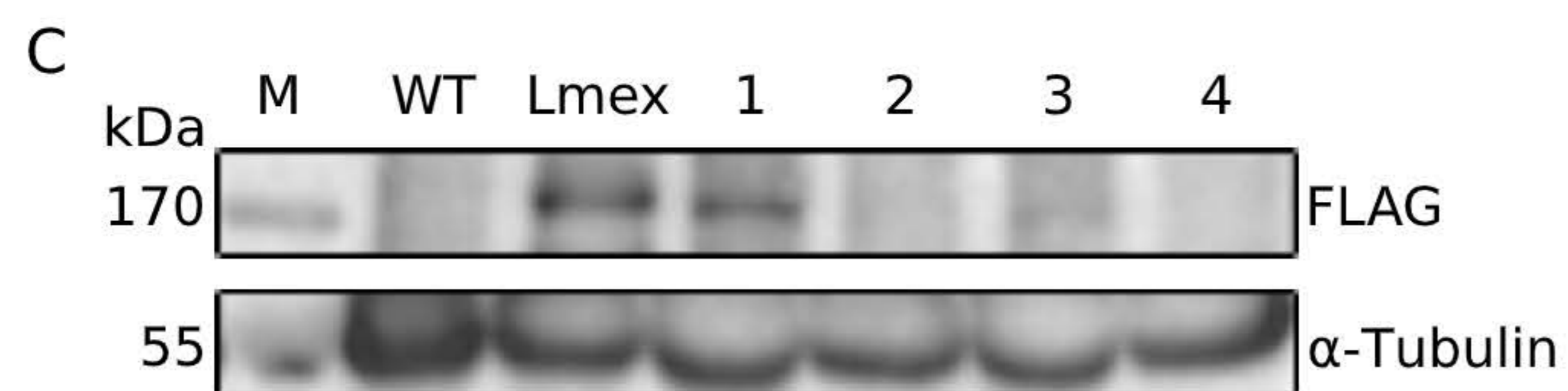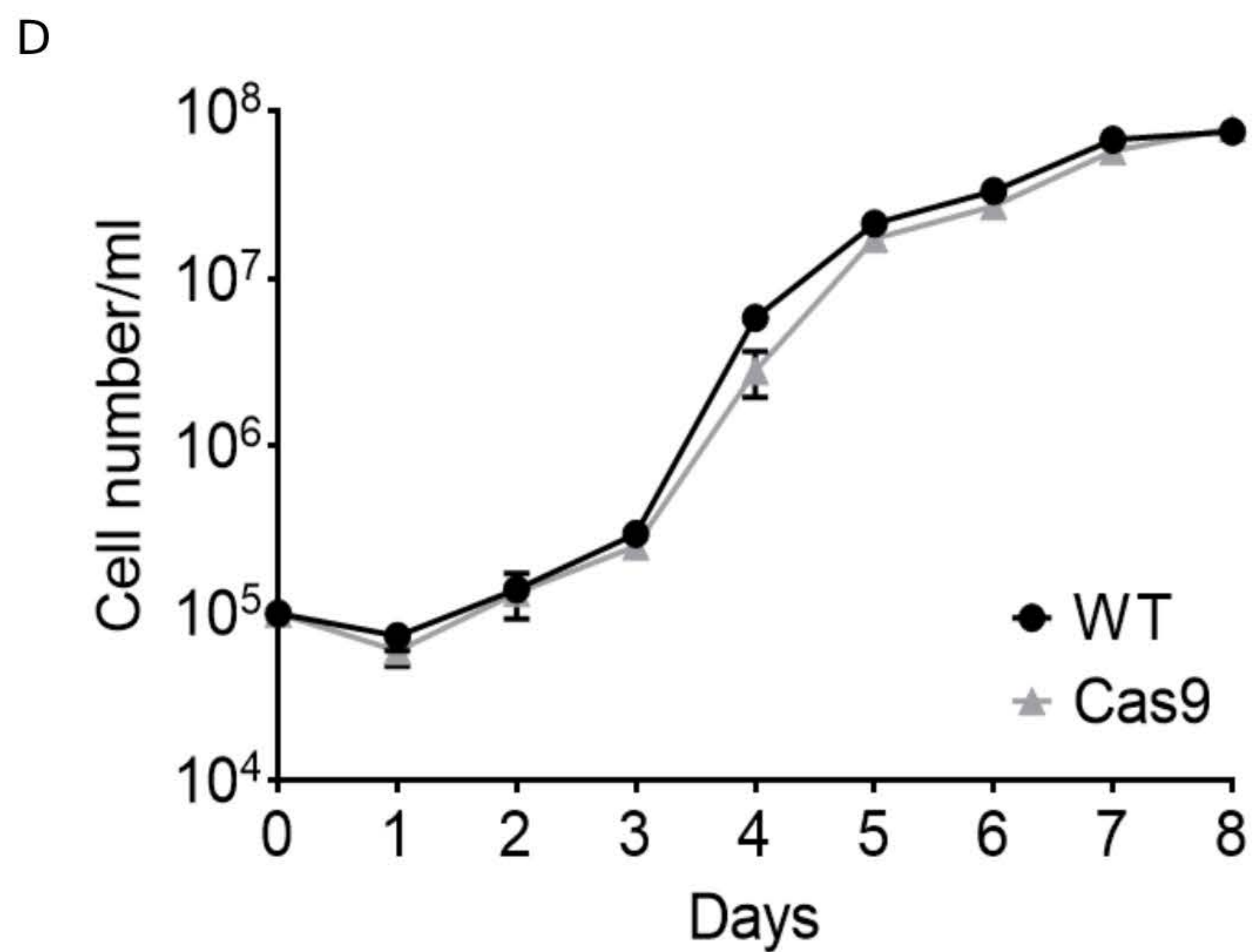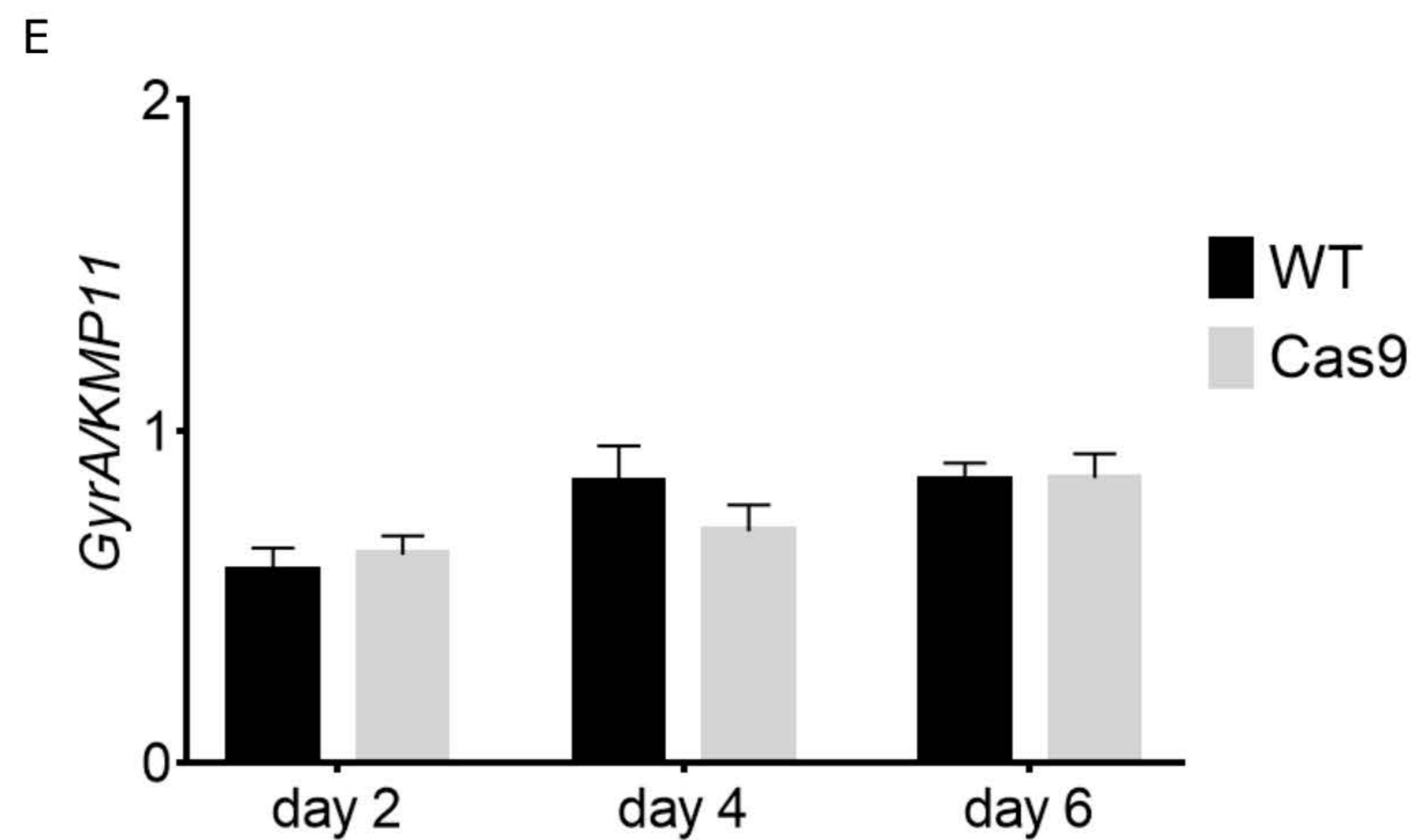

Supplement: FIG S9 [file mbio.01606-21-sf009.pdf]
